# Associations Between Sleep Deprivation, Circadian Gene Expression, Depressive Symptoms, and Psychomotor Performance—Preliminary Results

**DOI:** 10.3390/jcm15041331

**Published:** 2026-02-08

**Authors:** Marta Ditmer, Agata Gabryelska, Aleksandra Wojtera, Aleksandra Tarasiuk-Zawadzka, Agata Binienda, Szymon Turkiewicz, Filip Franciszek Karuga, Piotr Białasiewicz, Jakub Fichna, Dominik Strzelecki, Marcin Sochal

**Affiliations:** 1 Department of Sleep Medicine and Metabolic Disorders, Medical University of Lodz, 92-215 Lodz, Poland; 2 Department of Biochemistry, Medical University of Lodz, 92-215 Lodz, Poland; 3 Department of Affective and Psychotic Disorders, Medical University of Lodz, 92-213 Lodz, Poland

**Keywords:** sleep, sleep deprivation, circadian rhythm, clock

## Abstract

**Background:** Deprivation of sleep (DS) might affect mood and cognitive abilities, including psychomotor functions (PF). Molecular mechanisms underlying these effects remain unclear, though studies suggest that the circadian rhythm plays a role. **Methods:** Seventy participants underwent polysomnography (PSG) and DS. PF was evaluated using Bimanual Eye–Hand Coordination Test (BEHCT). Mood, PF, and clock gene expression (Circadian Locomotor Output Cycles Kaput (*CLOCK*), Brain and Muscle ARNT-Like 1 (*BMAL1*), Period Circadian Regulator 1 (*PER1*), Cryptochrome Circadian Regulator 1 (*CRY1*), Nuclear Receptor Subfamily 1 Group D Member 1 (*NR1D1*), and Neuronal PAS Domain Protein 2 (*NPAS2*)) were analyzed post-PSG and post-DS. Mood changes after DS classified participants as responders (RE) or non-responders (NR). **Results:** In NRs, but not REs, the BEHCT error count positively correlated with the expression of *BMAL1*, *CRY1*, *PER1*, *NR1D1* (R = 0.60, *p* = 0.002; R = 0.49, *p* = 0.018; R = 0.57, *p* = 0.023; and R = 0.53, *p* = 0.011, respectively), with *PER1* explaining its variability in 57.8% (b = 0.174, R^2^ = 0.578, F = 20.144, and *p* < 0.001). **Conclusions:** Obtained results suggest that altered clock gene expression may contribute to individual differences in mood and PF following DS.

## 1. Introduction

Deprivation of sleep (DS) refers to a deliberate or involuntary reduction in sleep duration. It exerts a wide range of biological effects, including increased oxidative stress and the disruption of immune system function [1,2]. Despite these largely adverse consequences, DS has been shown to exert a rapid antidepressant effect in a subset of individuals. More than six decades ago, Schulte, Pflug, and Tölle reported that acute DS could promptly alleviate depressive symptoms [3]. However, its clinical use as an adjunctive treatment remains limited due to the transient nature of this effect [4]. Meta-analyses suggest that approximately 50–80% of patients exhibit a positive mood response to DS, although this wide range may partly reflect the absence of standardized criteria for assessing mood improvement [4].

Beyond its effects on mood, DS is also associated with alterations in cognitive functioning. Evidence from population-based studies indicates that DS impairs cognitive performance, with the most pronounced effects observed in non-executive domains [5,6]. In contrast, tasks involving higher-order cognitive processes, such as sustained attention and working memory, appear to only be moderately affected [5,6]. This discrepancy may be explained by the nature of simpler tasks—such as the psychomotor vigilance test (PVT), which measures reaction time—that rely predominantly on attentional processes supported by a limited number of neural circuits, rendering them particularly susceptible to fatigue-related effects [7].

Importantly, changes in psychomotor performance following DS may be closely linked to mood alterations. Previous research has shown that individuals who respond to sleep deprivation tend to maintain or even improve cognitive and psychomotor performance after acute sleep loss, whereas non-responders demonstrate a decline in performance [8]. Notably, differences in vigilance appear to be strongly associated with the severity of depressive symptoms [8].

Despite these observations, the molecular mechanisms underlying the effects of DS on mental health remain incompletely understood. In our previous work, we proposed that clock genes may play a role in mediating the relationship between sleep disruption and mental health outcomes.

Circadian rhythms are regulated by a tightly controlled transcription–translation feedback loop involving core clock genes. Circadian Locomotor Output Cycles Kaput (*CLOCK*) and Brain and Muscle ARNT-Like Protein-1 (*BMAL1*) activate the transcription of the Period Circadian Regulator 1 (*PER1*) and Cryptochrome Circadian Regulator 1 (*CRY1*), while Nuclear Receptor Subfamily 1 Group D Member 1 (NR1D1) suppresses *CLOCK* and *BMAL1* activity. Additionally, Neuronal PAS Domain Protein 2 (NPAS2) can functionally substitute for CLOCK by forming a heterodimer with BMAL1 [9,10].

These clock genes play a central role in maintaining the balance between sleep, mood, and cognitive functioning, including psychomotor functions (PFs). Cognitive abilities such as attention, memory, and decision-making typically fluctuate with circadian rhythms, improving during periods of heightened circadian alertness [5,6]. In line with this, we previously demonstrated that DS reduces the expression of *CLOCK* and *BMAL1*, while increasing *PER1* expression [9]. Other human transcriptomic studies also indicate that DS can induce measurable changes in gene expression within a single night of sustained wakefulness [11]. Total DS has been shown to alter the rhythmicity, phase, and amplitude of core clock gene expression in peripheral blood cells, including phase shifts and amplitude attenuation of PER2 and BMAL1 rhythms, as well as a partial dissociation between clock gene expression and melatonin secretion [12,13].

The present secondary analysis aimed to examine the relationship between DS-induced changes in the mRNA expression of selected clock genes and psychomotor functioning [9].

## 2. Materials and Methods

### 2.1. Protocol

Individuals aged 18–35 years with a BMI of 20–30 kg/m^2^ were enrolled in the study. Exclusion criteria included pregnancy/lactation, chronic diseases, radio/chemotherapy, malignancies, except basal cell carcinoma, recent surgery, substance dependence, previously diagnosed sleep disorders, infection, and intercontinental travel within two weeks prior to enrolment.

The study included polysomnography (PSG) and DS. Participants gave informed consent and underwent physical examination before PSG. Polysomnographic (PSG) assessment comprised multiple physiological recordings, including electroencephalography (EEG) for cortical activity, chin and anterior tibialis electromyography (EMG) for muscle tone evaluation, and electrooculography (EOG) for eye movement detection. Oronasal airflow was measured using a thermistor, while snoring and body position were continuously monitored. Respiratory effort was assessed via piezoelectric belts placed around the chest and abdomen. Cardiac activity was recorded using a unipolar electrocardiogram (ECG), and arterial oxygen saturation was measured by pulse oximetry (SpO_2_) (Alice 6, Philips-Respironics). Sleep stages were manually scored in 30 s epochs in accordance with the guidelines of the American Academy of Sleep Medicine (AASM) [14].

A single episode of DS was performed under continuous actigraphic surveillance (GENEActiv Original, ActivInsights Ltd., Cambs, UK) approximately 2–4 weeks following polysomnography. On the evening of the scheduled procedure, participants were admitted to the Department of Sleep Medicine and Metabolic Disorders, where they were equipped with an actigraph and provided with detailed instructions regarding the DS protocol, including strict avoidance of daytime naps and psychoactive substances. Participants were permitted to remain in their own homes overnight. The DS period ended the next morning at approximately 08:00 AM. Actigraphy data were scored in accordance with American Academy of Sleep Medicine (AASM) recommendations [15]. Periods of sedentary behaviour were defined as epochs with a gravity-subtracted vector magnitude below 386 [16].

Venous blood samples (9 mL) were collected at two time points: in the morning following PSG and after the sleep deprivation night between 8 and 10 AM. Mood and PF assessments were conducted before and after each study phase by a trained rater using the Montgomery–Åsberg Depression Rating Scale (MADRS) and Bimanual Eye–Hand Coordination Test (BEHCT) (ELEKTROMET, Szczecin, Poland), respectively.

MADRS is a 10-item instrument for measuring depressive symptom severity across domains such as sadness, tension, and sleep, yielding total scores ranging from 0 to 60, with higher scores indicating greater symptom burden. A MADRS score > 7 was considered to be indicative of clinically relevant mild depression. Based on overnight changes in MADRS scores, participants were categorized as non-responders (NR; no improvement) or responders (RE; improvement or stable scores < 8).

BEHCT is designed to assess visuomotor coordination. During the task, participants trace a star-shaped outline using a stylus operated via two separate handwheels that control vertical and horizontal movements. The apparatus records performance metrics including task completion time, the number of deviations from the contour, and the cumulative duration spent outside the correct tracing path.

For each gene, MADRS, BEHCT error time, and error count change indices were calculated as the ratio of the post-sleep deprivation (post-SD) value to the post-polysomnography (post-PSG) value (Δ = post-SD/post-PSG).

The study protocol was approved by the Bioethics Committee of the Medical University of Lodz (reference number: RNN/302/20/KE). All procedures were performed per relevant guidelines, and regulations and complied with the Declaration of Helsinki principles. Informed consent was obtained from all participants’ guardians before their involvement in the study.

### 2.2. Molecular Analysis

Gene expression was assessed at two sampling points, corresponding to the morning following PSG and the morning after sleep deprivation. Total RNA was extracted using the TRIzol reagent (Invitrogen, Waltham, MA, USA), and its concentration was determined spectrophotometrically (NanoDrop Colibri, Titertek Berthold, Pforzheim, Germany). Complementary DNA (cDNA) was subsequently synthesized using the SuperScript™ IV First-Strand Synthesis System (Thermo Fisher Scientific, Carlsbad, CA, USA). Quantitative reverse transcription polymerase chain reaction (qRT-PCR) was performed on a Rotor-Gene™ 3000 real-time thermal cycler (Corbett Research, Mortlake, NSW, Australia). The PCR reaction mix contained TaqMan probes specific for *CLOCK*, *BMAL1*, *PER1*, *CRY1*, *NR1D1*, and *NPAS2*, the reference gene glyceraldehyde-3-phosphate dehydrogenase (*GAPDH*), nuclease-free water, a commercial master mix, and cDNA template. *GAPDH* was selected as the reference gene based on prior evidence demonstrating its stable expression under conditions of both total and paradoxical sleep deprivation in blood and brain tissue [17]. All samples were analyzed in triplicate, and cycle threshold (Ct) values were obtained for each replicate. Relative gene expression levels were calculated using ΔCt values and the 2^−ΔΔCt^ method.

### 2.3. Statistical Analysis

Statistical analysis was performed using Statistica 13.1PL (StatSoft, Tulsa, OK, USA), with significance set at *p* < 0.05. Data regarding gene expression were logarithmically transformed. Data distribution was assessed via the Shapiro–Wilk test. Parametric variables were analyzed with Student’s *t*-test and non-parametric variables with Wilcoxon signed-rank or Mann–Whitney U tests. Correlations were assessed using Spearman’s rank correlation coefficient. Statistical power was calculated based on Fisher’s Z transformation. Indices for each gene expression, BEHCT error time, error count, and MADRS score were defined as Δ = post-DS value/post-PSG value. Linear regression using a forward stepwise model was conducted to determine the impact of gene mRNA expression on ΔBEHCT error count.

## 3. Results

The primary study group included 74 participants. Three participants were excluded for missing BEHCT data and one for implausible results [9]. The final group included 37 women and 33 men; the median age was 24 years (interquartile range: 22–26). In the NR group (n = 26), MADRS scores were 2 (interquartile range, IQR 0–5) after PSG and 4 (IQR 1–8) after DS, resulting in a median ΔMADRS of −1 (IQR −3 to 0). In the RE group (n = 45), MADRS scores were 3.5 (IQR 1–6) after PSG and 2 (IQR 0–5) after DS, with a median ΔMADRS of 1 (IQR 0–4).

This study constitutes a secondary analysis, and further information on the study population has been reported previously [9,18].

There was no relationship between the results of the BEHCT and studied clock genes in the entire study group.

**Figure 1:**
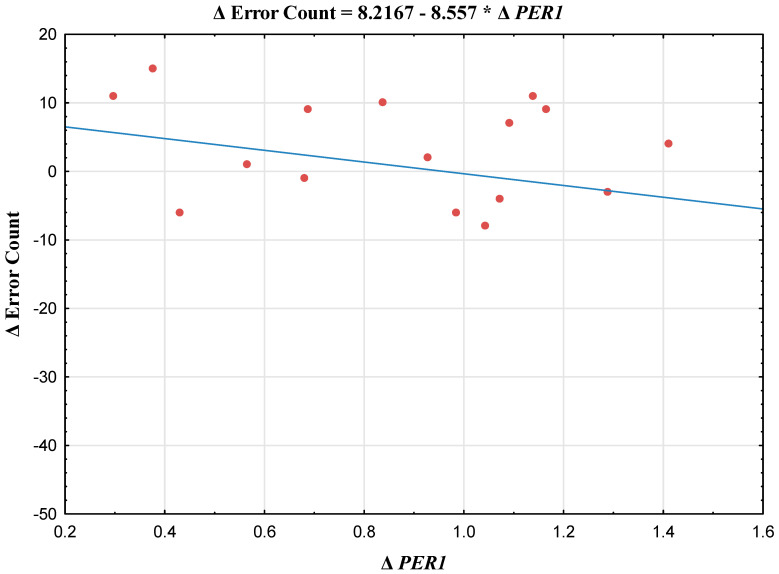
Scatterplot of Δ*PER1* expression versus ΔBimanual Eye–Hand Coordination Test Error Count in non-responders. Abbreviations: Period 1 (*PER1*).

In the NR group, positive correlations were observed between the ΔBEHCT error count (Δ = value after DS divided by value after PSG) and the following indices: Δ*BMAL1*, Δ*PER1* (a scatterplot illustrating this correlation is presented in Figure 1; additional scatterplots of the analyzed genes are available in the Supplementary Material (Figure S1–S53)), Δ*CRY1*, and Δ*NR1D1* (Table 1). 

Moreover, in this group, the ΔBEHCT error time was positively correlated with Δ*PER1*.

Task duration was not correlated with any of the studied clock genes. No correlations between clock gene expressions and BEHCT results were observed in the RE group. The linear regression model showed that, in NRs, 57.8% of the variability in the ΔBEHCT error count was explained by Δ*PER1* (b = 0.174, R^2^ = 0.578, F = 20.144, and *p* < 0.001).

## 4. Discussion

DS is well known to impair specific cognitive domains, particularly vigilance, while relatively preserving others, such as executive functioning [8]. Its effects on mood are also complex and vary substantially across individuals. Whereas some individuals experience a transient improvement in depressive symptoms, others exhibit a deterioration in emotional wellbeing [8]. Despite these observations, the relationship between individual mood responses to DS and accompanying changes in PF, as well as their molecular underpinnings, remains under-investigated. The present study sought to clarify these mechanisms by focusing on circadian rhythm genes, which play a role in both depressive symptomatology and cognitive performance [19,20].

In the present analysis, positive correlations between changes in gene expression (Δ*BMAL1*, Δ*PER1*, Δ*CRY1*, and Δ*NR1D1*) and error count were observed exclusively in NRs. In addition, in NRs, Δ*PER1* showed a positive association with the error time; both of the abovementioned parameters might be interpreted as a measure of psychomotor accuracy. Moreover, change in the expression levels of this gene was able to explain 57.8% of ΔBEHCT error count in NRs, suggesting its importance as a potential molecular correlate between DS, mood, and cognitive faculties.

To the best of our knowledge, this is the first study to examine the interactions between DS, mood, clock gene expression, and PF. Previous studies investigated these factors independently. Groeger et al. demonstrated that humans homozygous for the longer variant of the *PER3* gene performed worse on executive function tests following DS compared to those with the shorter variant; mood or mRNA expression were not evaluated [21]. In our previous report, DS reduced *CLOCK* and *BMAL1* expression, increased *PER1*, and affected cognition based on mood response; REs maintained or improved performance, whereas NRs declined [8].

Other authors also highlighted the relationship between clock genes and depression. Polymorphisms of *CLOCK* and several other circadian rhythm genes were associated with this disorder [22,23]. Furthermore, glycogen synthase kinase-3, which phosphorylates core clock proteins, may mediate therapeutic antidepressant effects [22,24]. Similarly, studies support the notion that circadian fluctuations influence executive functions [25].

A potential mechanistic link between circadian clock regulation, sleep, cognitive function, and mood may involve interactions between circadian rhythm genes and neurotrophins (NTs). NTs are essential for maintaining central nervous system integrity and play a critical role in synaptic plasticity, neurogenesis, and gliogenesis—processes long implicated in mood improvement following DS [26,27]. Research in this field has predominantly focused on the brain-derived neurotrophic factor (BDNF), the most abundant neurotrophin in the central nervous system [28,29]. By contrast, other neurotrophins, including glial cell line-derived neurotrophic factor (GDNF), neurotrophin-3 (NT3), and neurotrophin-4 (NT4), remain relatively under-investigated. Evidence also indicates that NT synthesis and activity are linked to circadian regulation. The low-affinity NT receptor p75NTR is directly regulated by the CLOCK:BMAL1 transcriptional complex, highlighting a molecular interface between circadian timing and neurotrophic signalling [30]. Moreover, Turkiewicz et al. demonstrated a correlation between *PER1* mRNA and *BDNF* expression [31]. Together, these findings point to PER1’s role as a potential molecular correlate linking DS-induced mood changes and cognitive outcomes; however, its link to NTs requires more detailed investigation.

Giese et al. reported that depressed individuals who exhibited mood improvement following sleep deprivation had higher BDNF levels compared with non-responders [32]. In contrast, in the previous report, DS was associated with a reduction in BDNF mRNA expression across all participants without corresponding changes in circulating BDNF protein levels [18]. Notably, serum NT4 concentrations increased in all participants, potentially compensating for the reduction in BDNF, given the functional redundancy of these neurotrophins in mammals [26]. Additionally, divergent GDNF responses were observed between groups, with decreased GDNF protein levels in non-responders and reduced GDNF mRNA expression in responders [18].

Although GDNF is less extensively studied in the context of cognition than BDNF, emerging evidence suggests a potential role in cognitive processes. Chencheng et al. demonstrated that GDNF may improve cognitive function in a mouse model of Parkinson’s disease through the modulation of dopamine transporter activity [33]. Similarly, studies in aged murine models indicate that GDNF can promote synaptic plasticity [34]. Furthermore, De Souza et al. proposed that GDNF may serve as a prodromal biomarker for early subjective cognitive complaints, although this association appeared to be sex-specific and limited to males with depression [35]. Collectively, these findings highlight the need for further investigation into the role of GDNF in mood-related cognitive outcomes, particularly with respect to specific cognitive domains and their interaction with the circadian clock. Additionally, the relationship between NT4 and circadian rhythm regulation warrants further study, ideally in the context of cognitive performance and its interplay with BDNF signalling.

This exploratory study has several limitations. Mood and psychomotor functioning were assessed using a limited set of instruments, and actigraphy has reduced sensitivity for detecting brief naps. Gene expression was measured only at the mRNA level without assessment of corresponding protein concentrations, which limits the insight into downstream functional mechanisms, although this approach is appropriate for short-term DS protocols.

The absence of direct circadian phase markers (e.g., melatonin or cortisol) prevents disentangling DS-related effects from circadian misalignment, and some observed associations may partly reflect phase shifts rather than DS, per se.

Mood response was categorized using a binary MADRS classification to ensure adequate group sizes. While this facilitated the identification of broad response patterns, it likely oversimplified emotional changes and obscured more subtle or heterogeneous effects.

Importantly, the modest sample size reinforces the exploratory nature of both the study and the regression analyses, which were limited to a small number of predictors and should be interpreted cautiously. Moreover, due to the observational design, causal inferences cannot be drawn; associations involving PER1 expression therefore represent preliminary correlational findings rather than evidence of a mediating role.

Overall, our results suggest that the increased expression of *BMAL1*, *CRY1*, *PER1*, and *NR1D1* genes is associated with impaired PF in individuals who do not experience mood improvement after DS. Among these, PER1 appears particularly relevant and may serve as a potential molecular correlate linking DS-induced mood changes and cognitive outcomes.

## Figures and Tables

**Table 1:** Correlations between the clock gene expressions and the Bimanual Eye–Hand Coordination Test (BEHCT) results.

		All			NR			RE		
		n	r	*p*	n	r	*p*	n	r	*p*
Δ*CLOCK*	Δ Task Duration	66	−0.02	0.843	23	−0.09	0.687	43	−0.01	0.933
	Δ Error Time	66	−0.06	0.644	23	0.20	0.366	43	−0.13	0.415
	Δ Error Count	66	−0.02	0.864	23	0.34	0.114	43	−0.17	0.265
Δ*BMAL1*	Δ Task Duration	67	−0.02	0.858	25	0.08	0.701	42	−0.10	0.528
	Δ Error Time	66	0.14	0.277	24	0.28	0.187	42	0.04	0.785
	Δ Error Count	66	0.17	0.174	**24**	**0.60**	0.002, 0.888 *	42	−0.03	0.866
Δ*PER1*	Δ Task Duration	46	0.16	0.275	16	−0.04	0.880	30	0.27	0.145
	Δ Error Time	46	0.18	0.232	**16**	**0.67**	0.004, 0.833 *	30	−0.02	0.934
	Δ Error Count	46	0.10	0.514	**16**	**0.57**	0.023, 0.647 *	30	−0.09	0.639
Δ*CRY1*	Δ Task Duration	67	0.02	0.849	24	−0.02	0.933	43	0.04	0.812
	Δ Error Time	66	0.06	0.621	23	0.30	0.162	43	−0.07	0.656
	Δ Error Count	66	0.09	0.463	**23**	**0.49**	0.018, 0.669 *	43	−0.10	0.535
Δ*NR1D1*	Δ Task Duration	61	−0.01	0.940	22	0.20	0.368	39	−0.10	0.527
	Δ Error Time	61	<0.01	0.988	22	0.34	0.125	39	−0.13	0.444
	Δ Error Count	61	0.03	0.837	**22**	**0.53**	0.011, 0.729 *	39	−0.13	0.413
Δ*NPAS2*	Δ Task Duration	46	−0.06	0.705	16	−0.02	0.940	30	−0.10	0.596
	Δ Error Time	46	−0.03	0.841	16	0.01	0.974	30	−0.01	0.966
	Δ Error Count	46	−0.04	0.790	16	−0.06	0.812	30	−0.01	0.949

* Study power calculation. Study power was calculated using Fisher’s Z transformation. Abbreviations: Circadian Locomotor Output Cycles Kaput (*CLOCK*), Brain and Muscle ARNT-Like Protein-1 (*BMAL1*), Period Circadian Regulator 1 (*PER1*), Cryptochrome Circadian Regulator 1 (*CRY1*), non-respondents (NR), Nuclear Receptor Subfamily 1 Group D Member 1 (*NR1D1*), Neuronal PAS Domain Protein 2 (*NPAS2*), and respondents (RE). Indices for each gene expression, BEHCT error time, and error count were defined as Δ = post-DS value/post-PSG value. Bold values indicate statical significance.

## Data Availability

The raw data supporting the conclusions of this article will be made available by the authors on request.

## Supplementary Materials

The following supporting information can be downloaded at: https://www.mdpi.com/article/10.3390/jcm15041331/s1, Additional scatterplots of analyzed genes can be found in the supplementary material.
