# Supplementary material for: Associations Between Sleep Deprivation, Circadian Gene Expression, Depressive Symptoms, and Psychomotor Performance—Preliminary Results
Source: J Clin Med. 2026 Feb 8;15(4):1331. doi: 10.3390/jcm15041331 (PMC12940950; doi:10.3390/jcm15041331)
Supplement: Supplementary file 1 [file jcm-15-01331-s001.zip › jcm-4082903-supplementary.pdf]

Figure S1. Scatterplot of Δ*CLOCK* expression versus ΔBimanual Eye-Hand Coordination Test Task Duration in the entire study group.

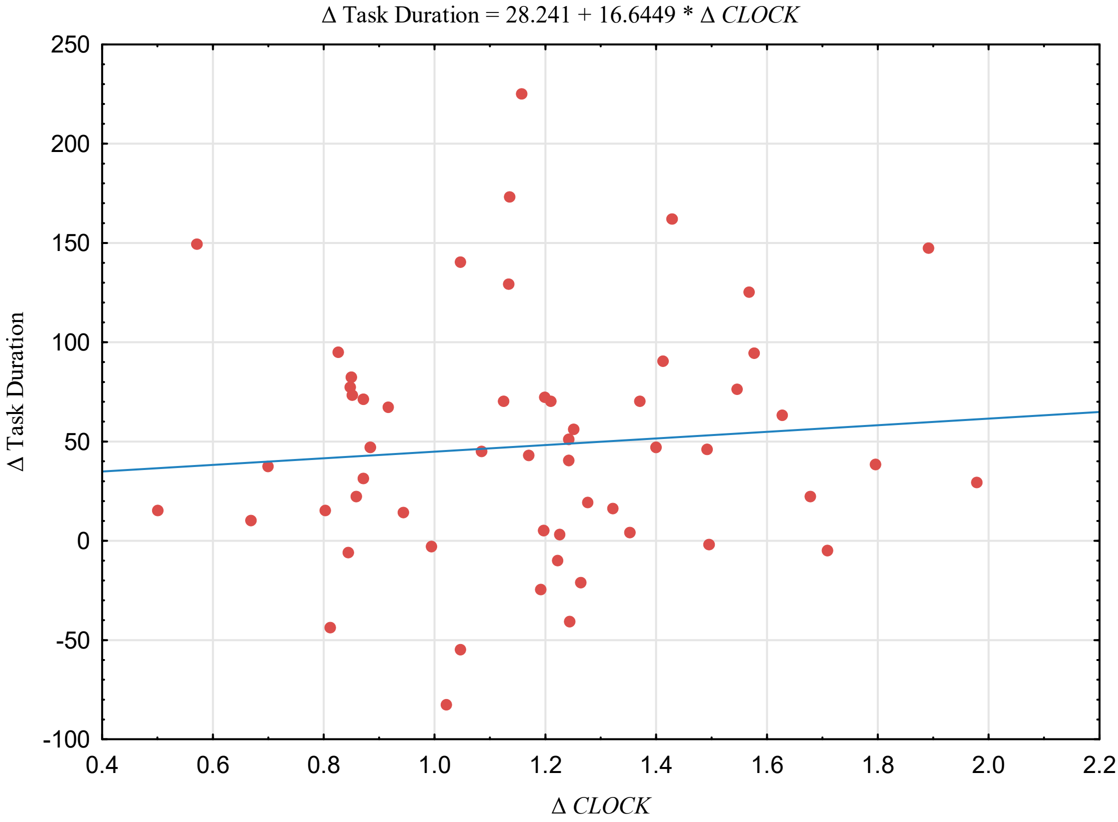

Abbreviations: CLOCK - Circadian Locomotor Output Cycles Kaput.

Figure S2. Scatterplot of Δ*CLOCK* expression versus ΔBimanual Eye-Hand Coordination Test Error Time in the entire study group.

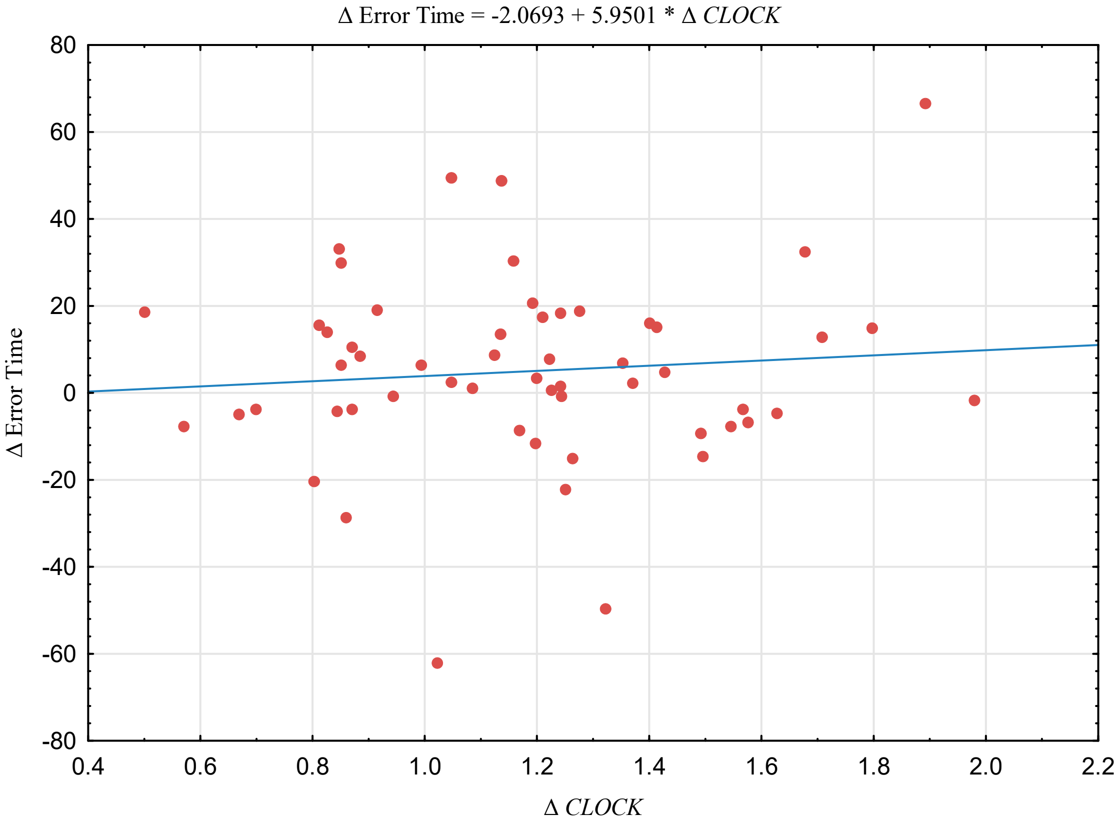

Abbreviations: CLOCK - Circadian Locomotor Output Cycles Kaput.

Figure S3. Scatterplot of Δ*CLOCK* expression versus ΔBimanual Eye-Hand Coordination Test Error Count in the entire study group.

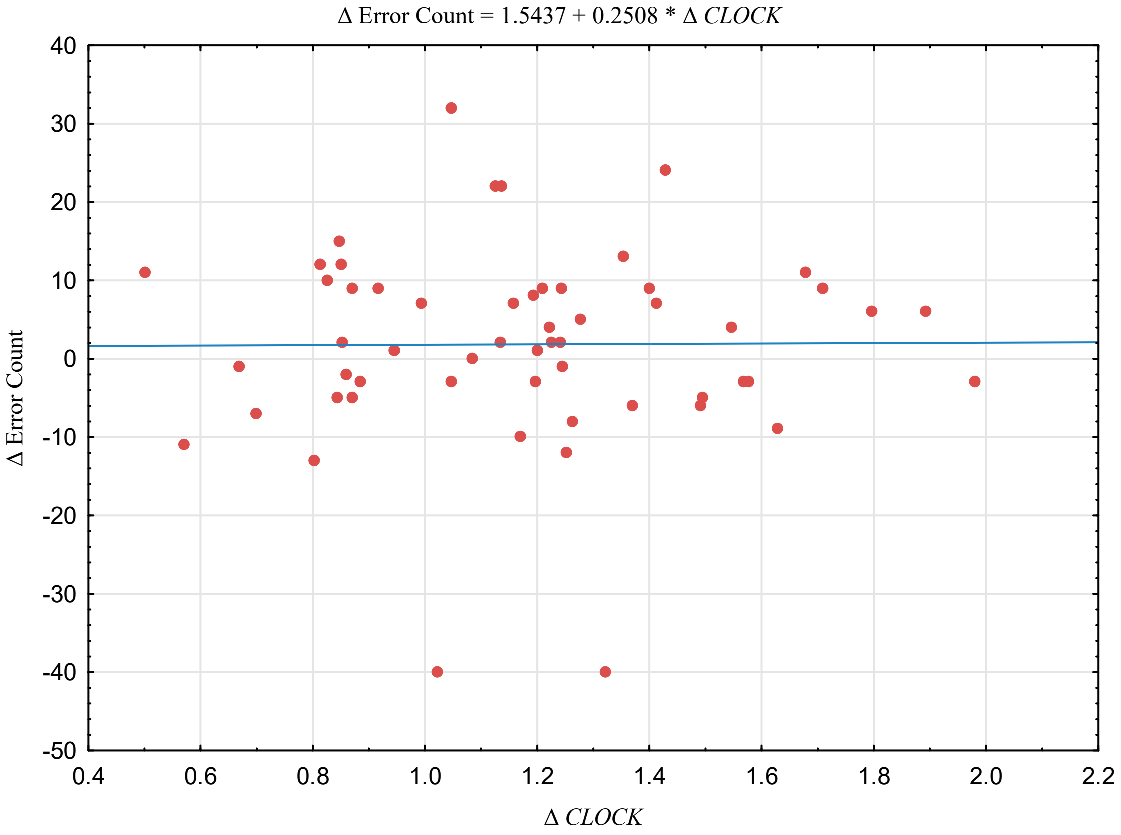

Abbreviations: CLOCK - Circadian Locomotor Output Cycles Kaput.

Figure S4. Scatterplot of Δ*CLOCK* expression versus ΔBimanual Eye-Hand Coordination Test Task Duration in Responders.

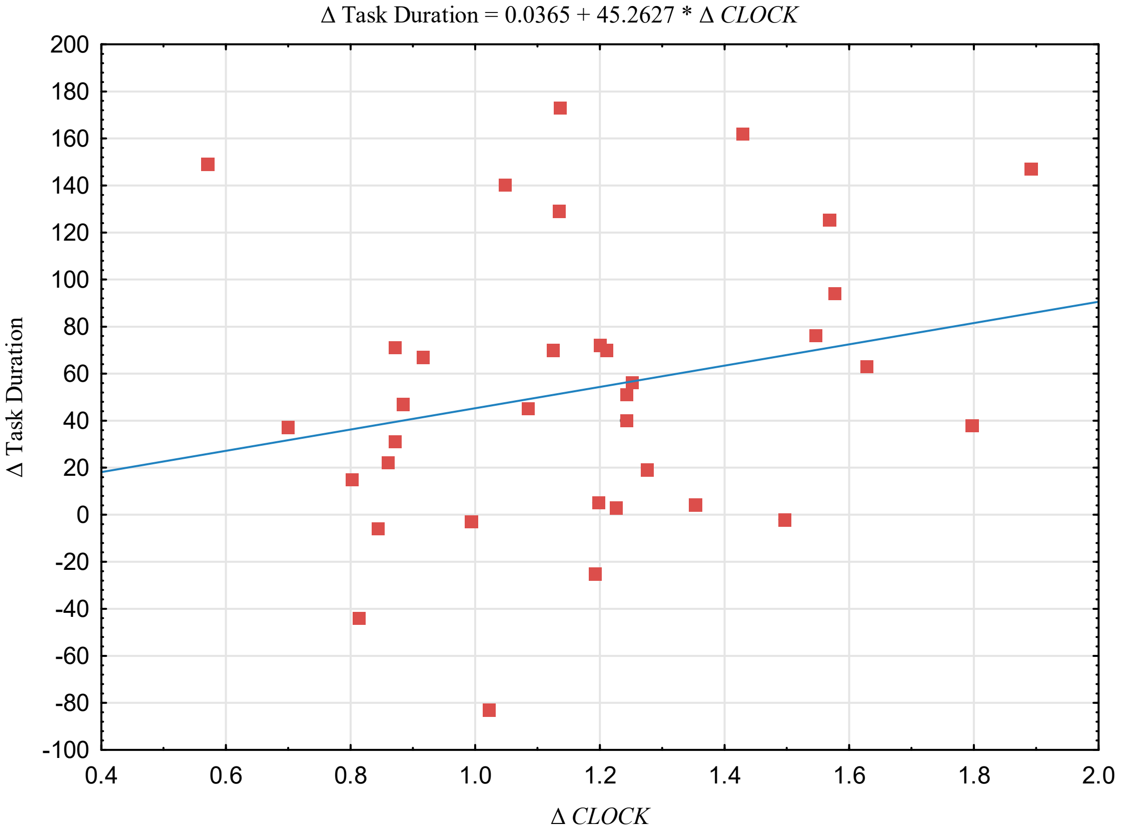

Abbreviations: CLOCK - Circadian Locomotor Output Cycles Kaput.

Figure S5. Scatterplot of Δ*CLOCK* expression versus ΔBimanual Eye-Hand Coordination Test Error Time in Responders.

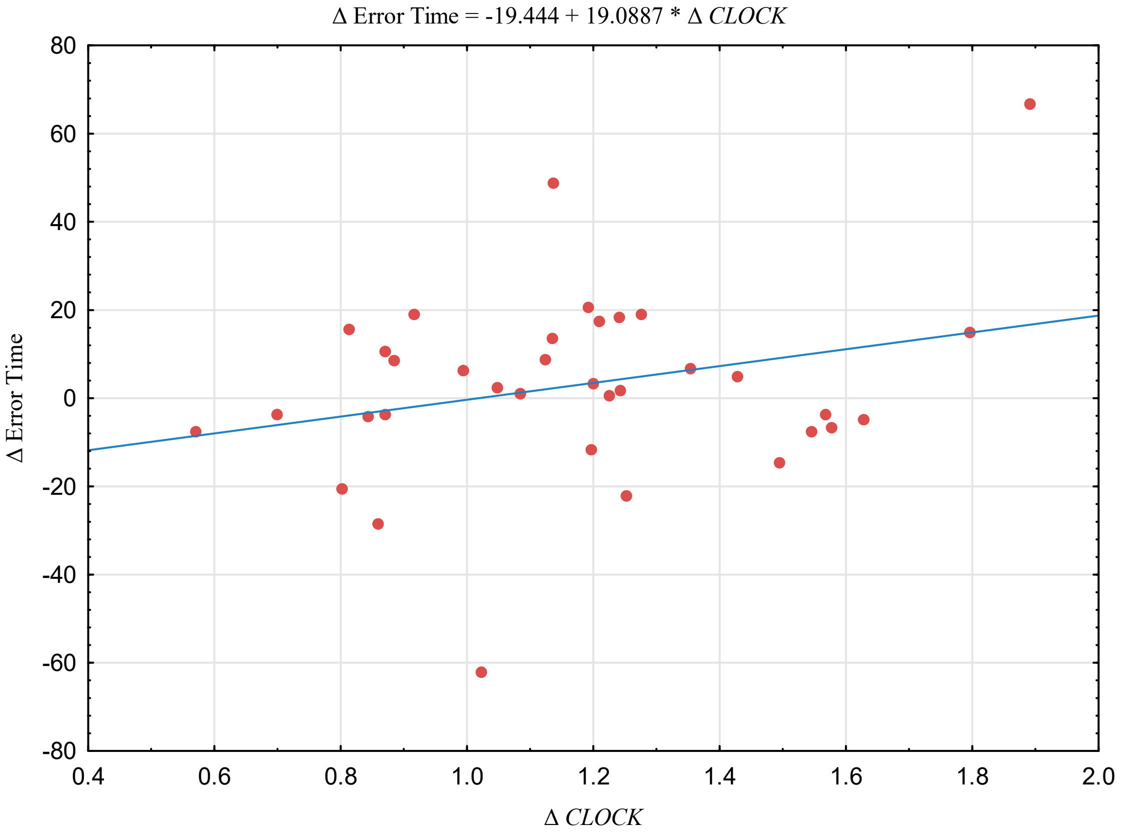

Abbreviations: CLOCK - Circadian Locomotor Output Cycles Kaput.

Figure S6. Scatterplot of Δ*CLOCK* expression versus ΔBimanual Eye-Hand Coordination Test Error Count in Responders.

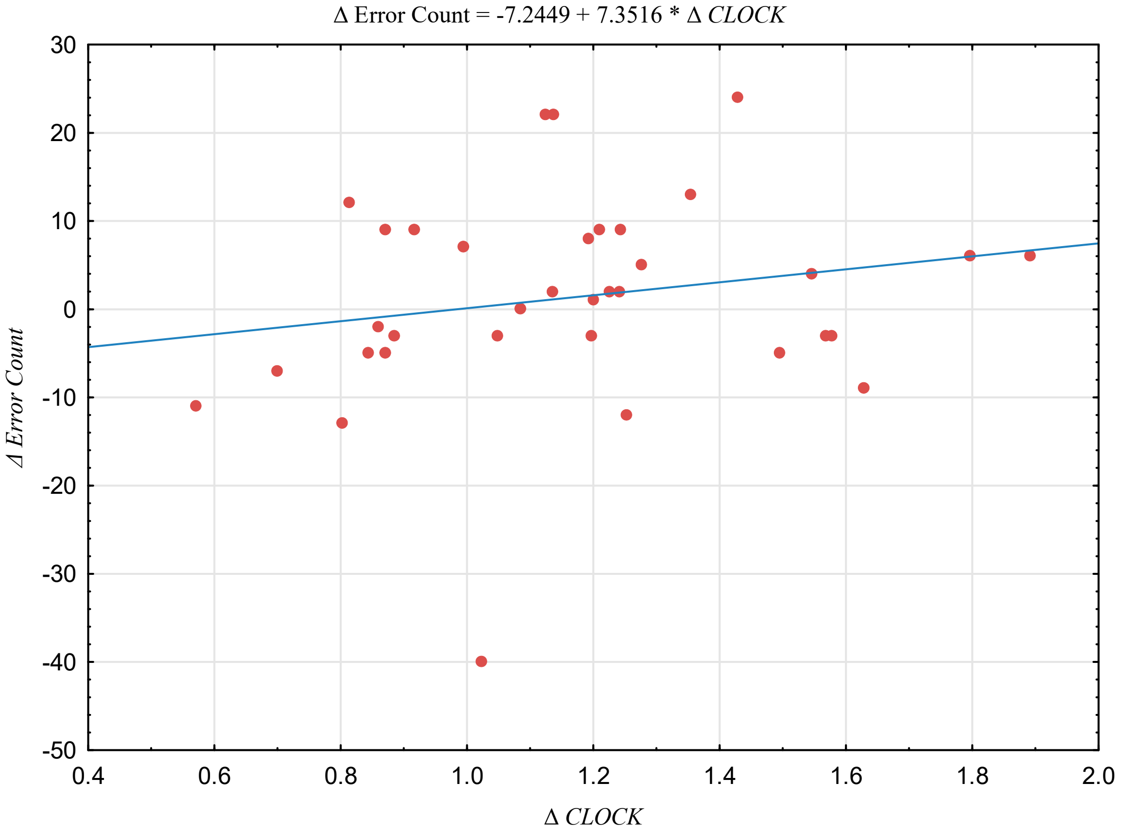

Abbreviations: CLOCK - Circadian Locomotor Output Cycles Kaput.

Figure S7. Scatterplot of Δ*CLOCK* expression versus ΔBimanual Eye-Hand Coordination Test Task Duration in Non-Responders.

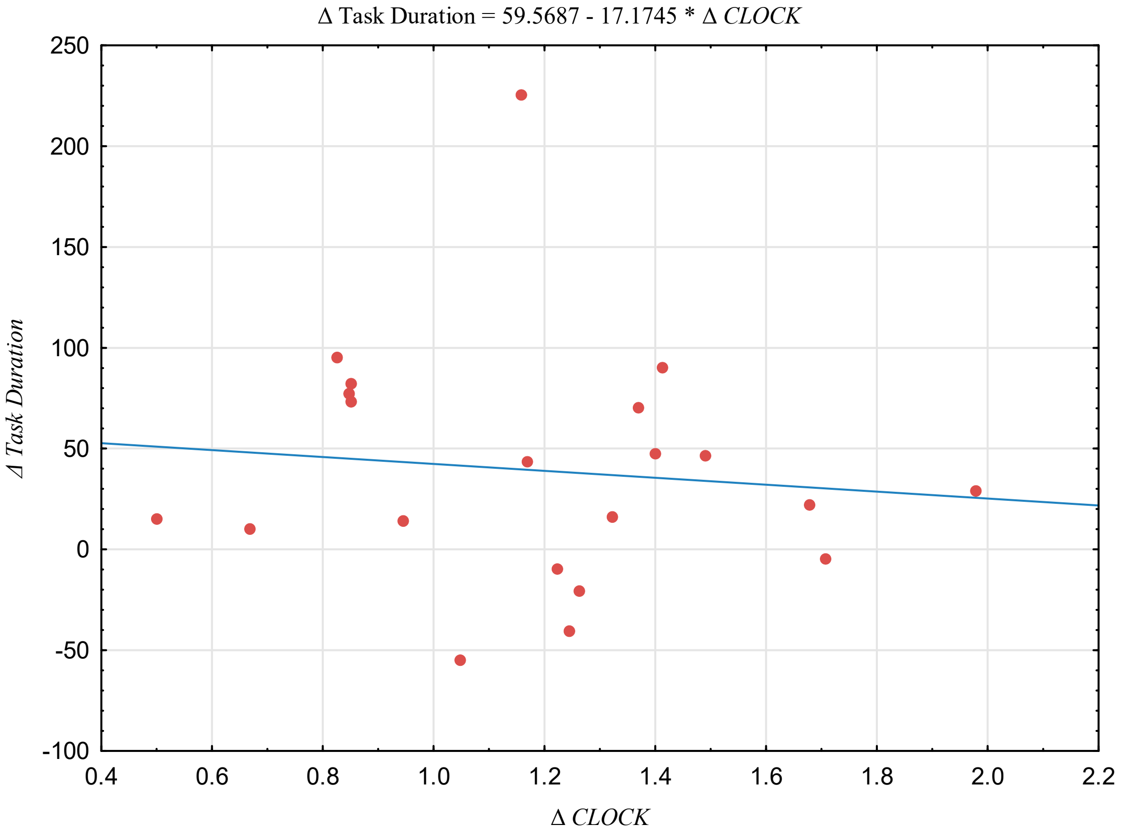

Abbreviations: CLOCK - Circadian Locomotor Output Cycles Kaput.

Figure S8. Scatterplot of Δ*CLOCK* expression versus ΔBimanual Eye-Hand Coordination Test Error Time in Non-Responders.

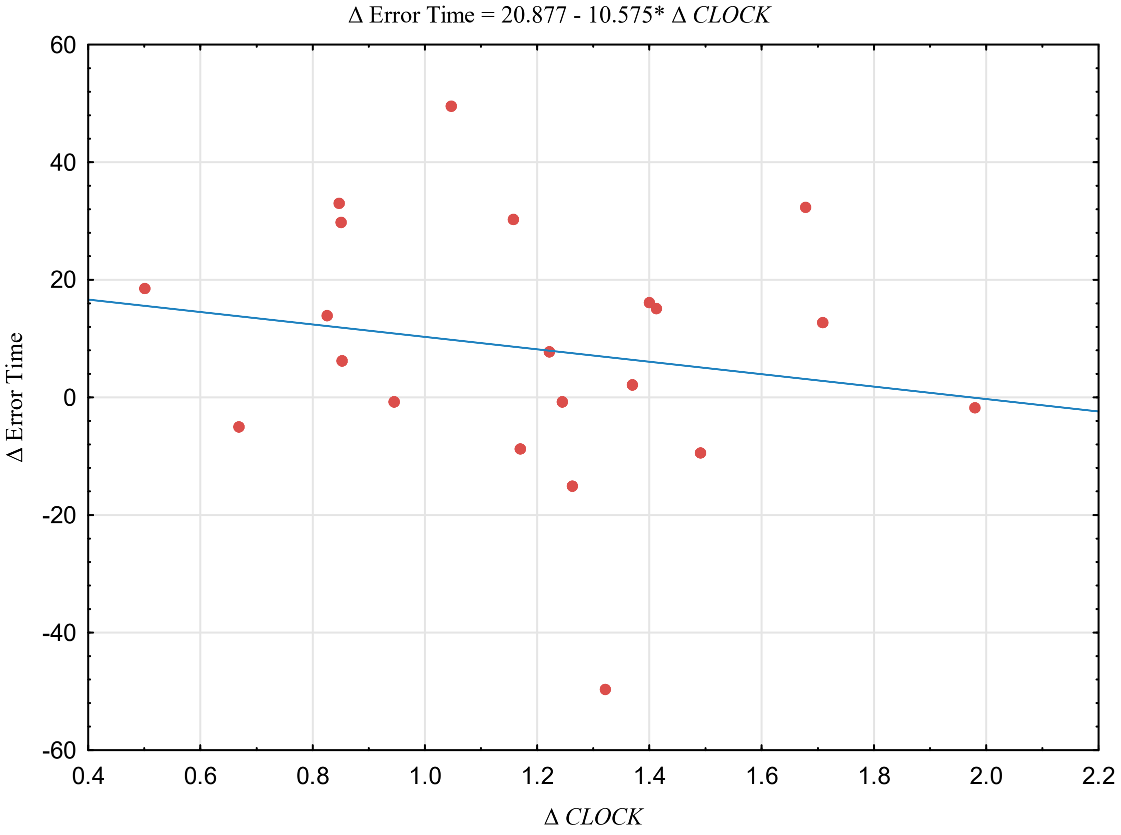

Abbreviations: CLOCK - Circadian Locomotor Output Cycles Kaput.

Figure S9. Scatterplot of Δ*CLOCK* expression versus ΔBimanual Eye-Hand Coordination Test Error Count in Non-Responders.

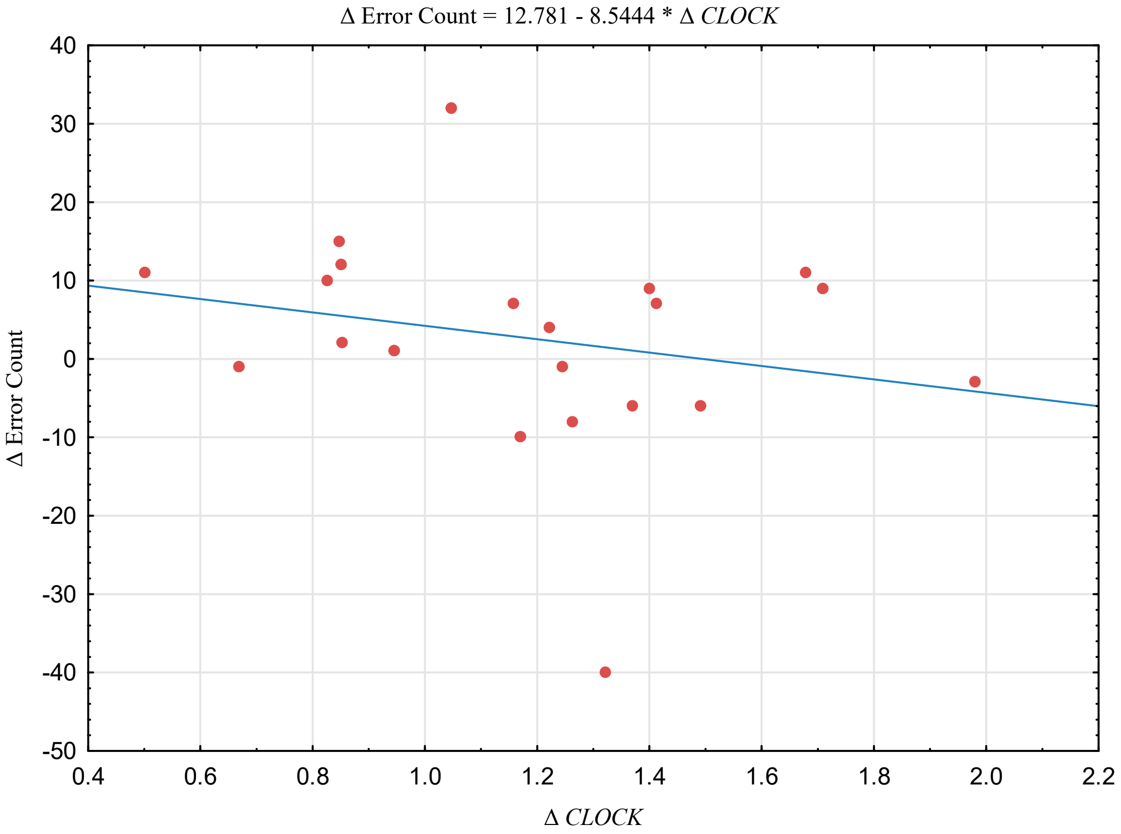

Abbreviations: CLOCK - Circadian Locomotor Output Cycles Kaput.

Figure S10. Scatterplot of Δ*BMAL1* expression versus ΔBimanual Eye-Hand Coordination Test Task Duration in the entire study group.

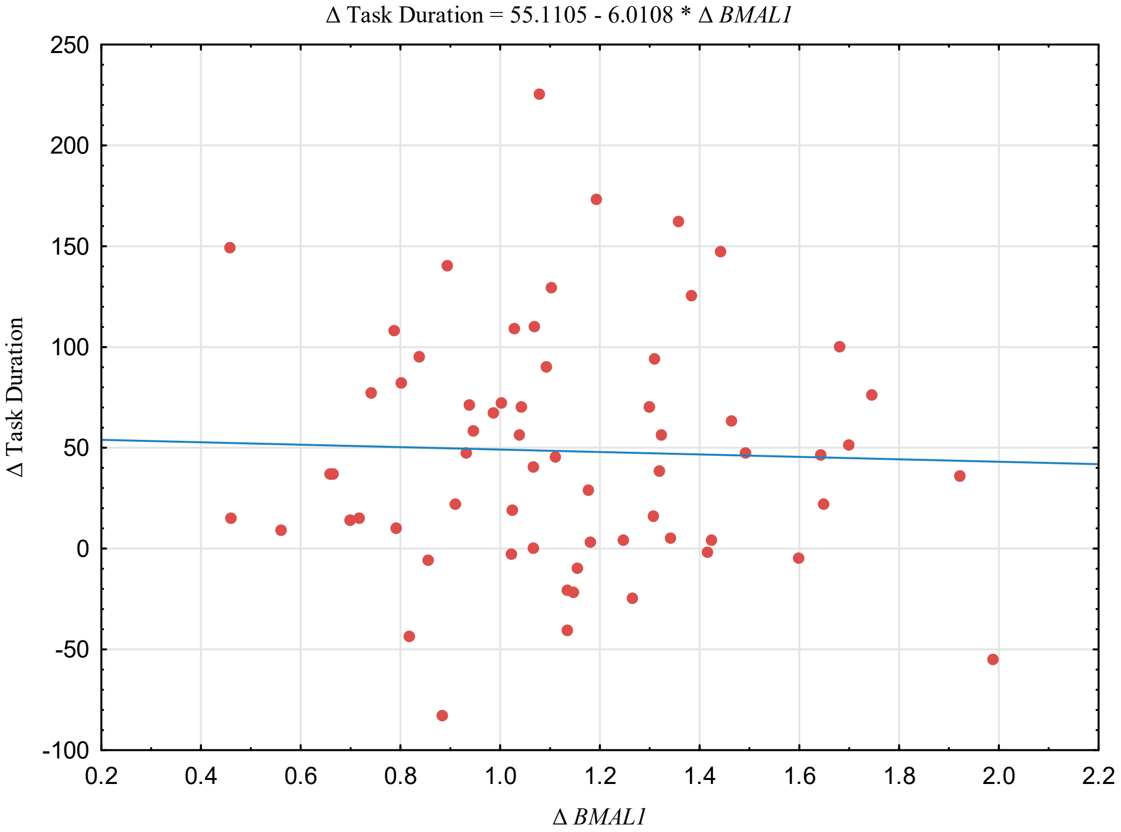

Abbreviations: BMAL1 - Brain and Muscle ARNT-Like 1.

Figure S11. Scatterplot of Δ*BMAL1* expression versus ΔBimanual Eye-Hand Coordination Test Error Time in the entire study group.

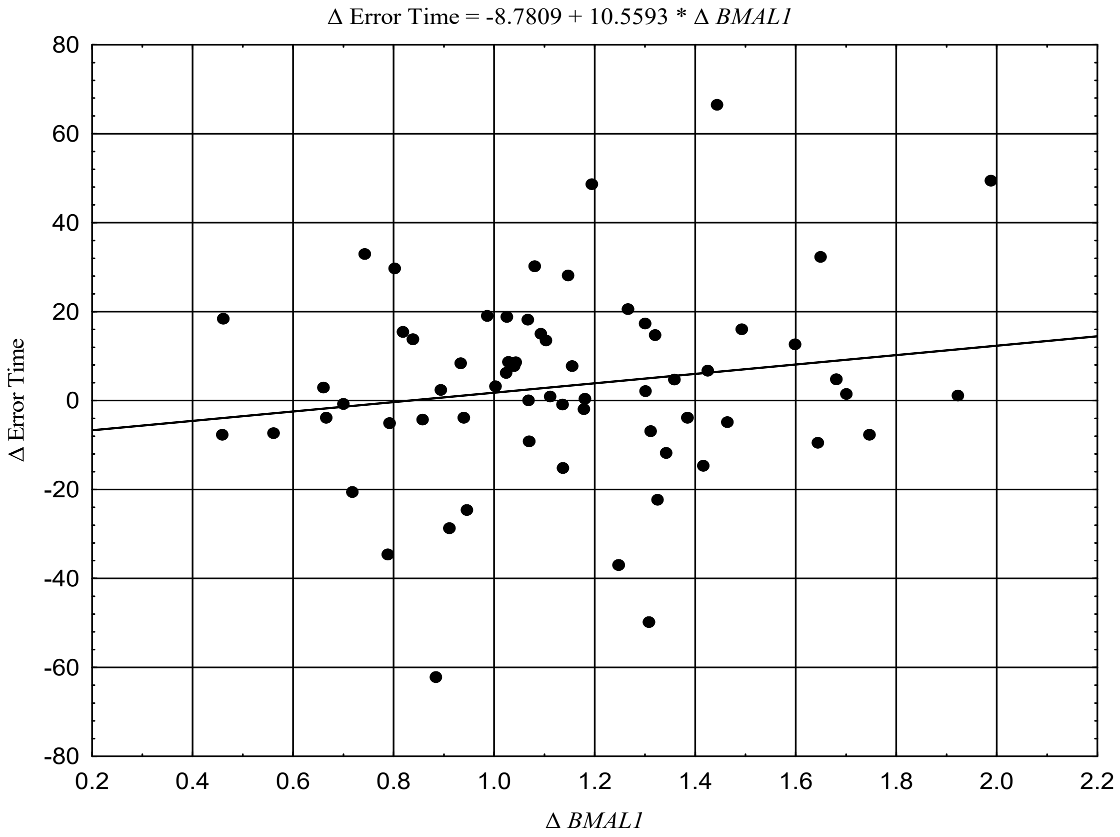

Abbreviations: BMAL1 - Brain and Muscle ARNT-Like 1.

Figure S12. Scatterplot of Δ*BMAL1* expression versus ΔBimanual Eye-Hand Coordination Test Error Count in the entire study group.

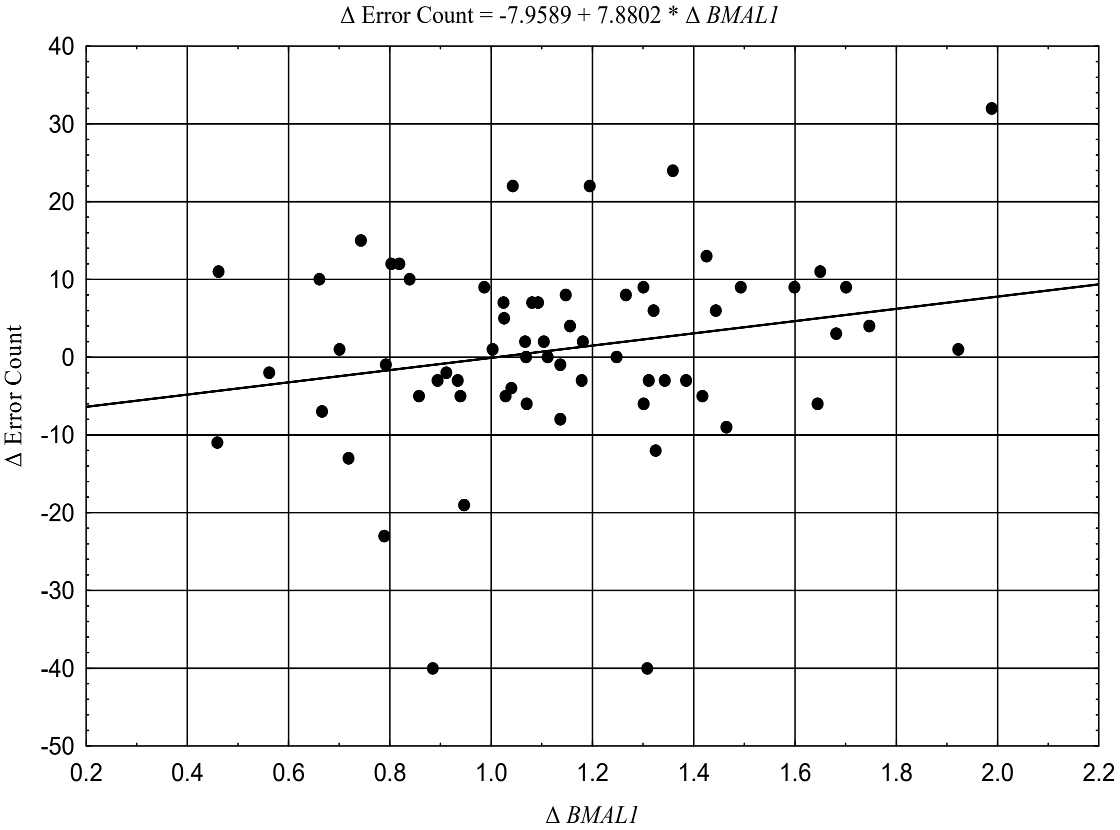

Abbreviations: BMAL1 - Brain and Muscle ARNT-Like 1.

Figure S13. Scatterplot of Δ*BMAL1* expression versus ΔBimanual Eye-Hand Coordination Test Task Duration in Responders.

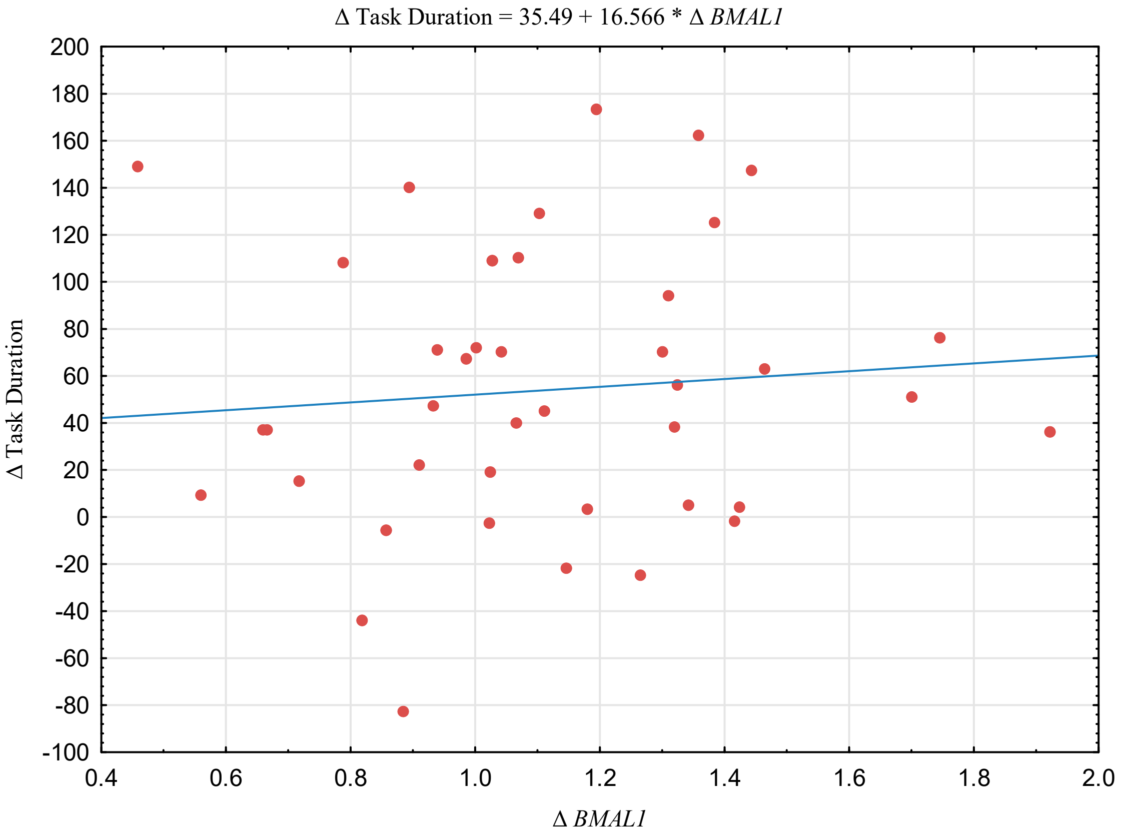

Abbreviations: BMAL1 - Brain and Muscle ARNT-Like 1.

Figure S14. Scatterplot of Δ*BMAL1* expression versus ΔBimanual Eye-Hand Coordination Test Error Time in Responders.

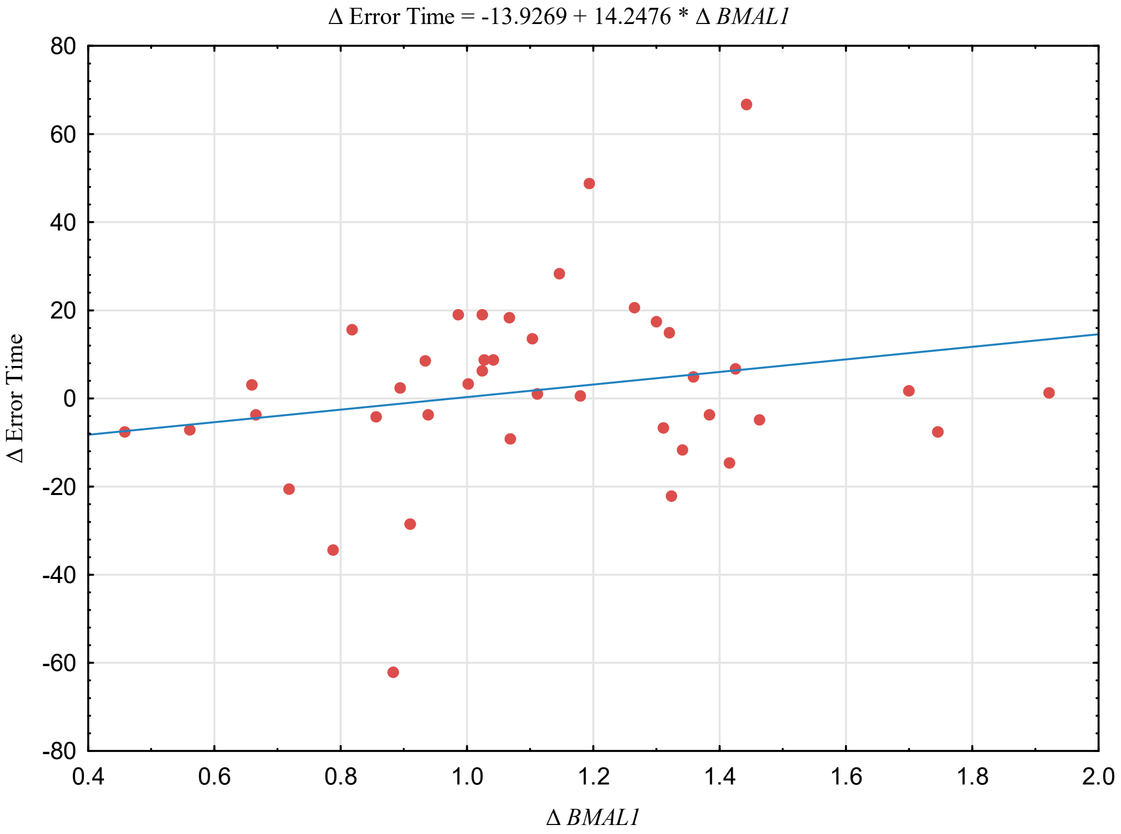

Abbreviations: BMAL1 - Brain and Muscle ARNT-Like 1.

Figure S15. Scatterplot of Δ*BMAL1* expression versus ΔBimanual Eye-Hand Coordination Test Error Count in Responders.

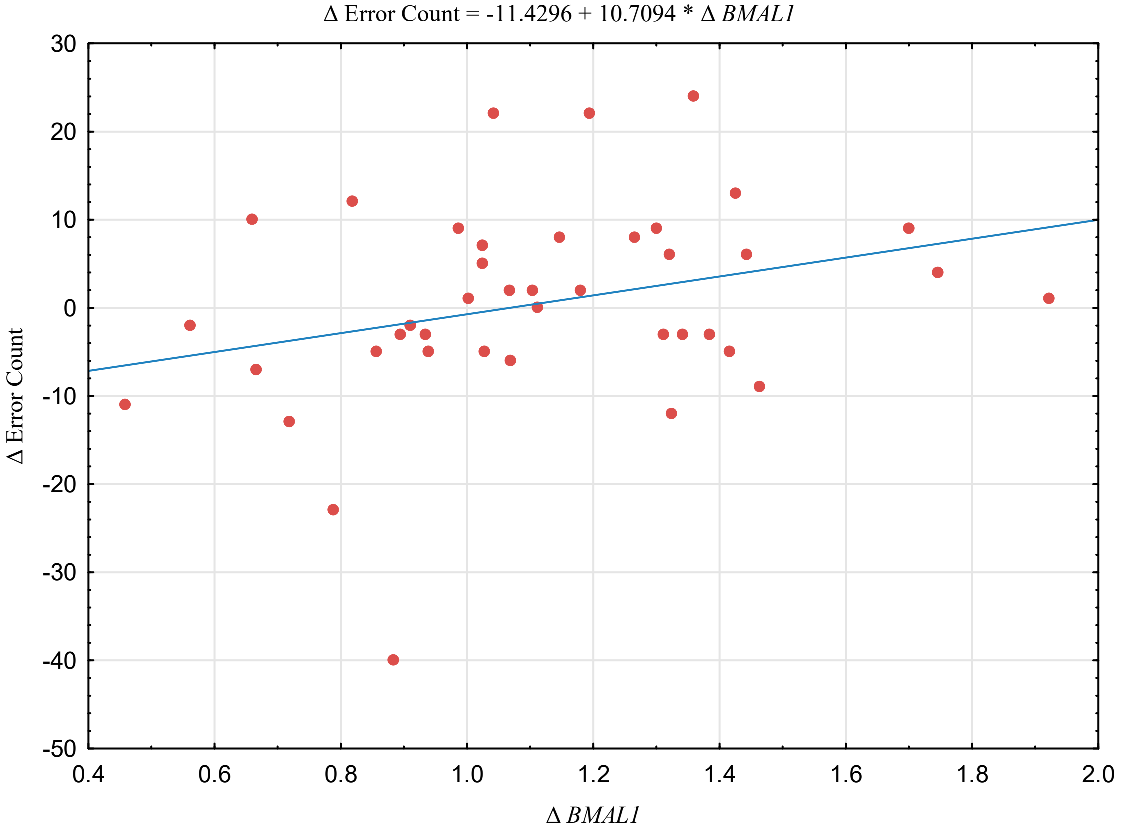

Abbreviations: BMAL1 - Brain and Muscle ARNT-Like 1.

Figure S16. Scatterplot of Δ*BMAL1* expression versus ΔBimanual Eye-Hand Coordination Test Task Duration in Non-Responders.

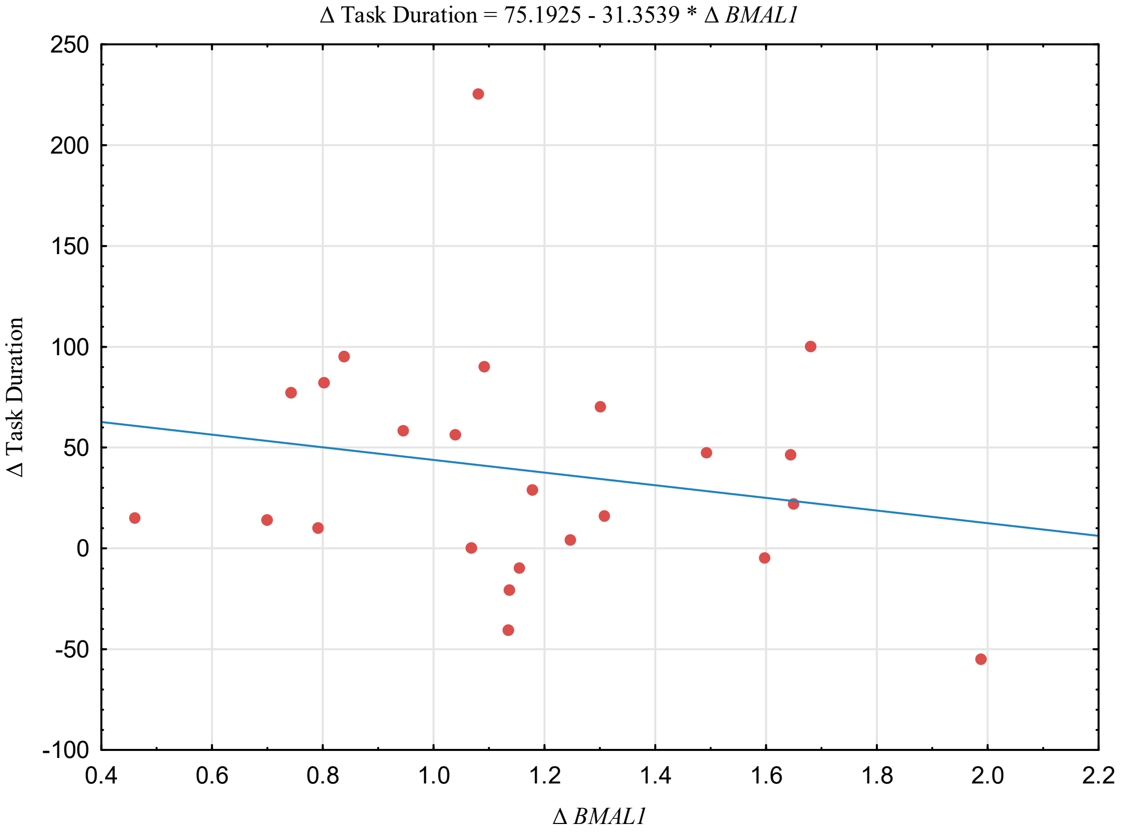

Abbreviations: BMAL1 - Brain and Muscle ARNT-Like 1.

Figure S17. Scatterplot of Δ*BMAL1* expression versus ΔBimanual Eye-Hand Coordination Test Error Time in Non-Responders.

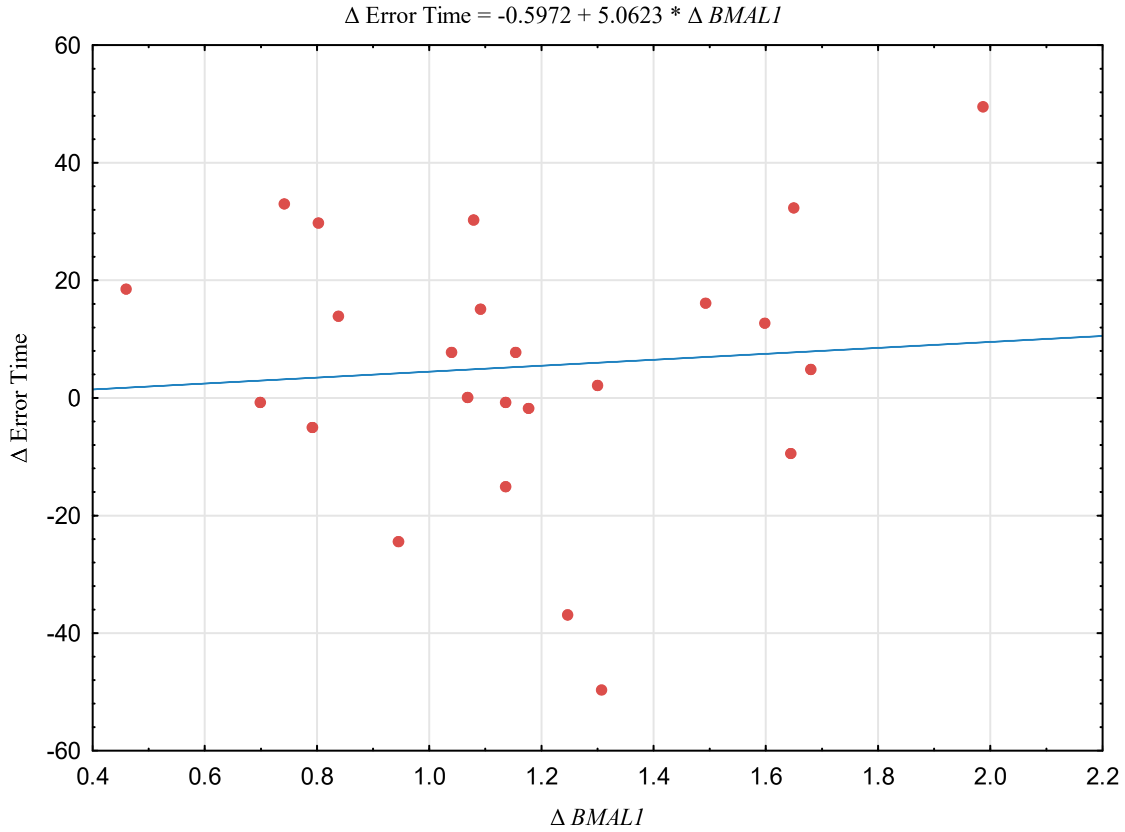

Abbreviations: BMAL1 - Brain and Muscle ARNT-Like 1.

Figure S18. Scatterplot of Δ*BMAL1* expression versus ΔBimanual Eye-Hand Coordination Test Error Count in Non-Responders.

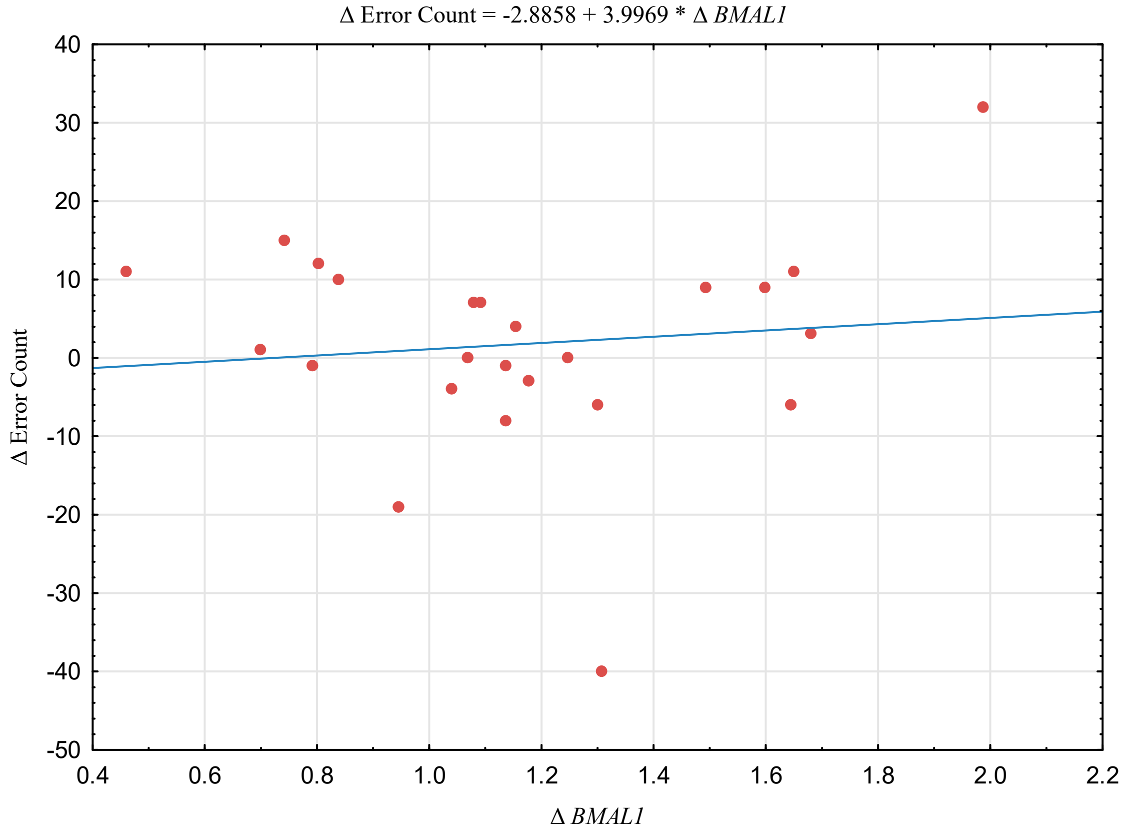

Abbreviations: BMAL1 - Brain and Muscle ARNT-Like 1.

Figure S19. Scatterplot of Δ*PER1* expression versus ΔBimanual Eye-Hand Coordination Test Task Duration in the entire study group.

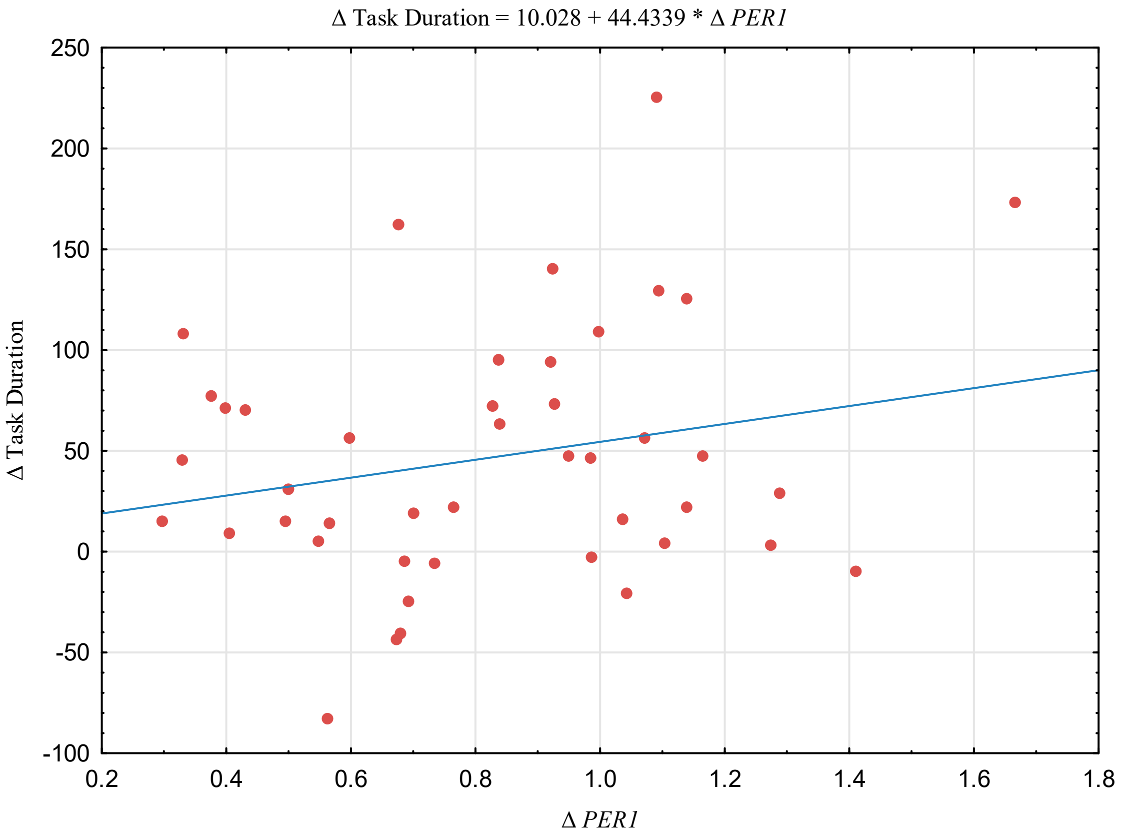

Abbreviations: PER1 - Period circadian regulator 1.

Figure S20. Scatterplot of Δ*PER1* expression versus ΔBimanual Eye-Hand Coordination Test Error Time in the entire study group.

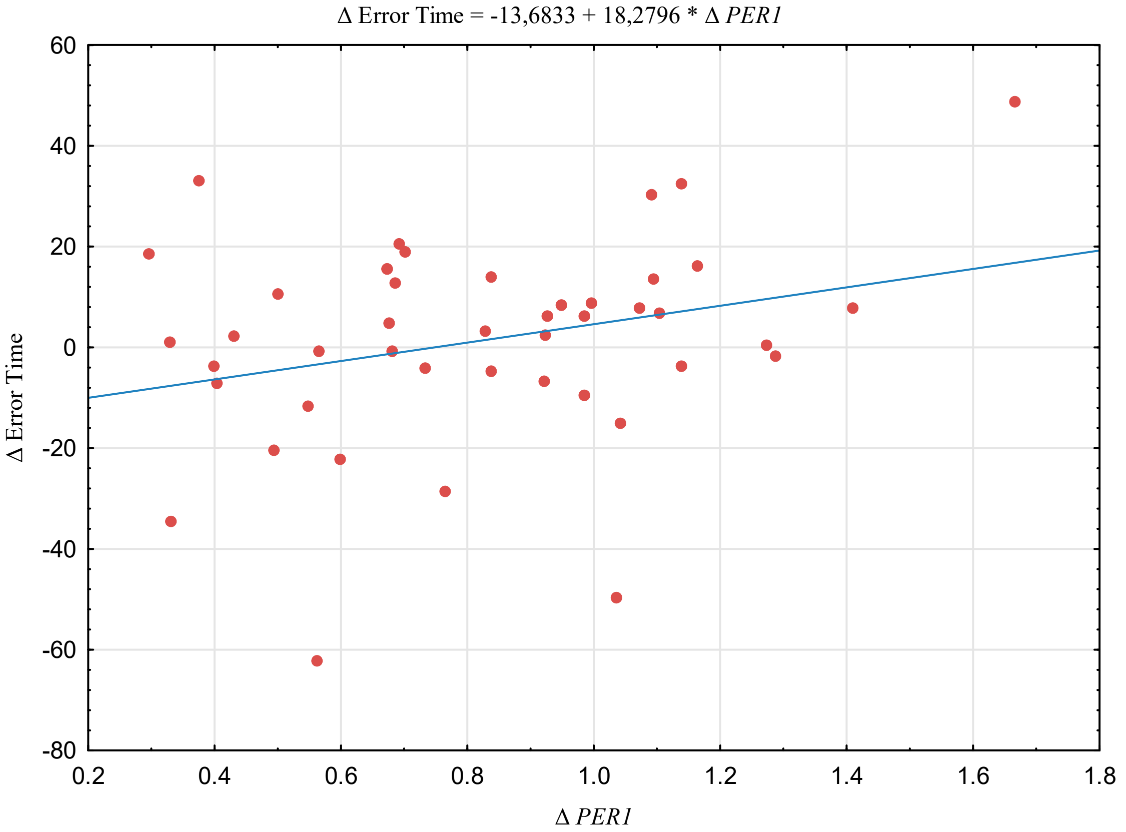

Abbreviations: PER1 - Period circadian regulator 1.

Figure S21. Scatterplot of Δ*PER1* expression versus ΔBimanual Eye-Hand Coordination Test Error Count in the entire study group.

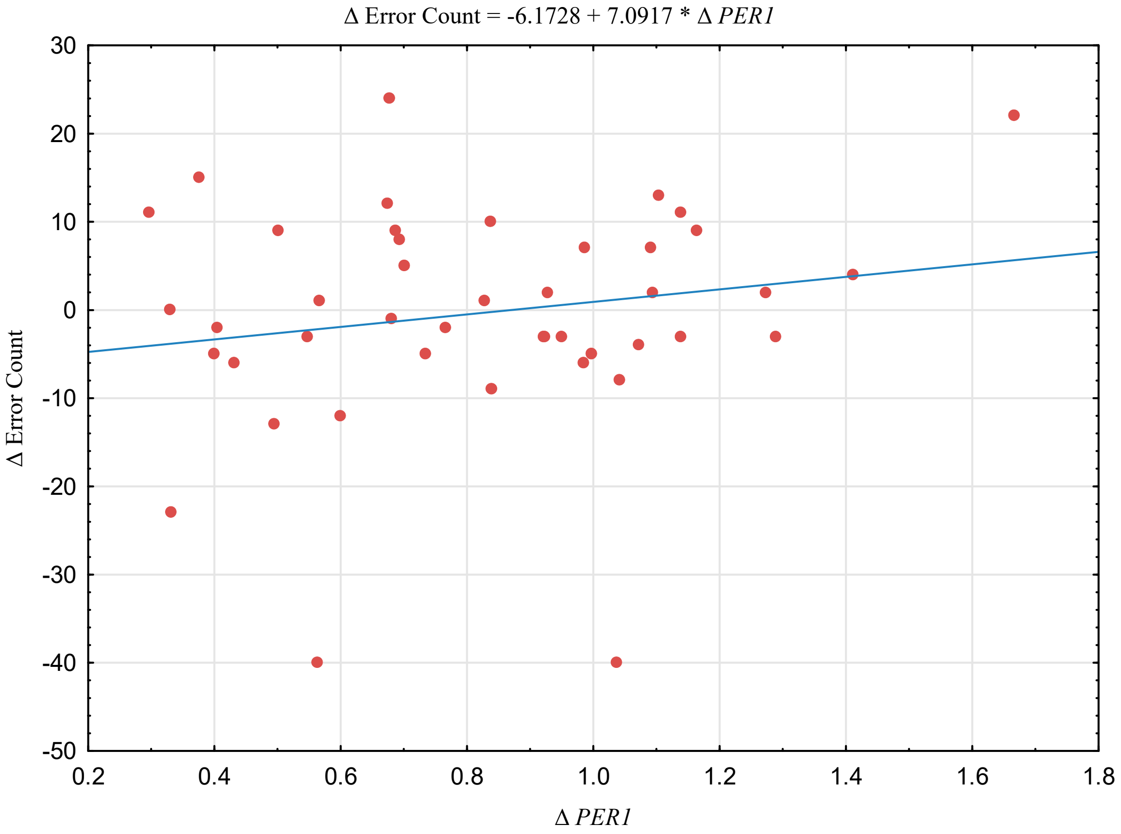

Abbreviations: PER1 - Period circadian regulator 1.

Figure S22. Scatterplot of Δ*PER1* expression versus ΔBimanual Eye-Hand Coordination Test Task Duration in Responders.

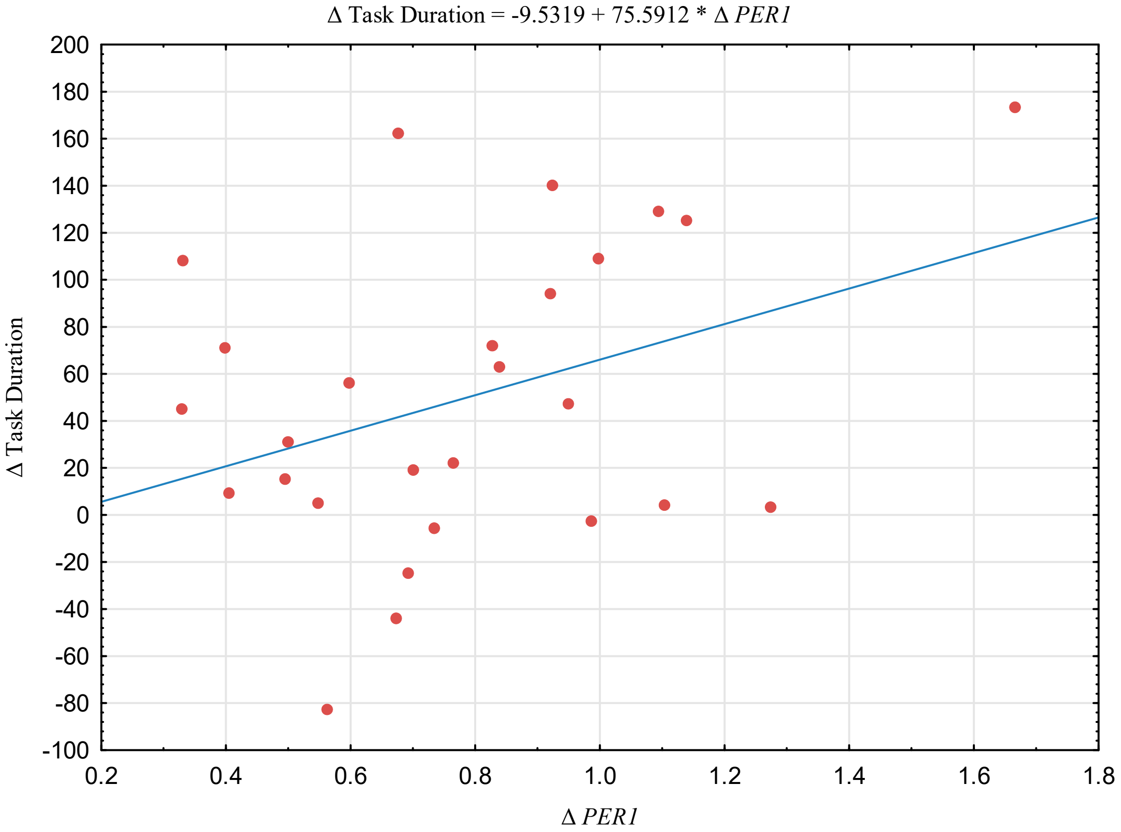

Abbreviations: PER1 - Period circadian regulator 1.

Figure S23. Scatterplot of Δ*PER1* expression versus ΔBimanual Eye-Hand Coordination Test Error Time in Responders.

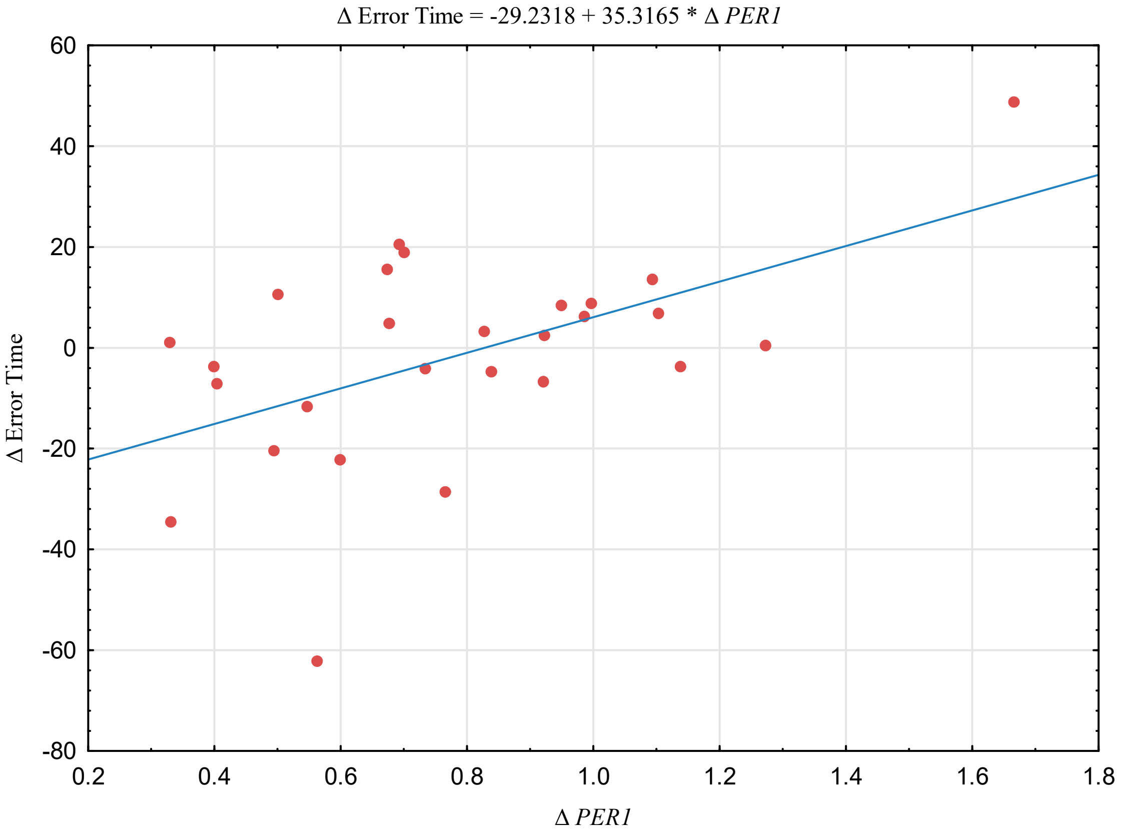

Abbreviations: PER1 - Period circadian regulator 1.

Figure S24. Scatterplot of Δ*PER1* expression versus ΔBimanual Eye-Hand Coordination Test Error Count in Responders.

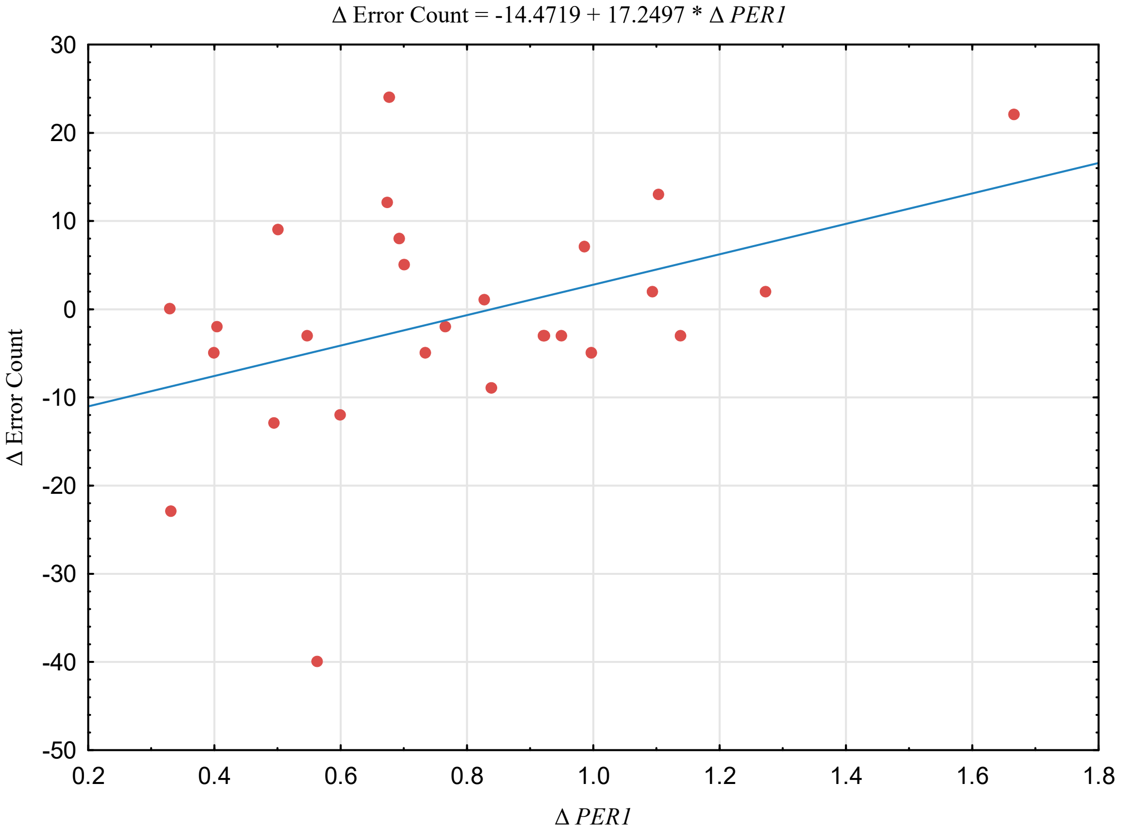

Abbreviations: PER1 - Period circadian regulator 1.

Figure S25. Scatterplot of Δ*PER1* expression versus ΔBimanual Eye-Hand Coordination Test Task Duration in Non-Responders.

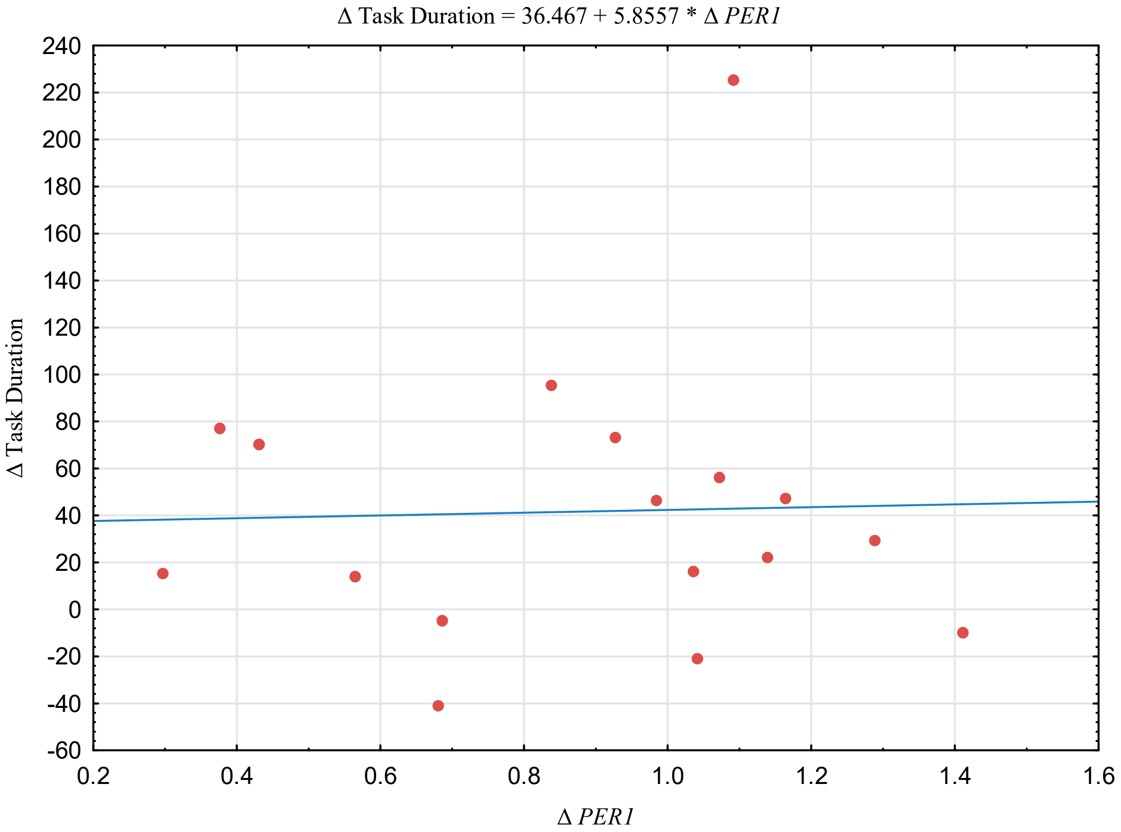

Abbreviations: PER1 - Period circadian regulator 1.

Figure S26. Scatterplot of Δ*PER1* expression versus ΔBimanual Eye-Hand Coordination Test Error Time in Non-Responders.

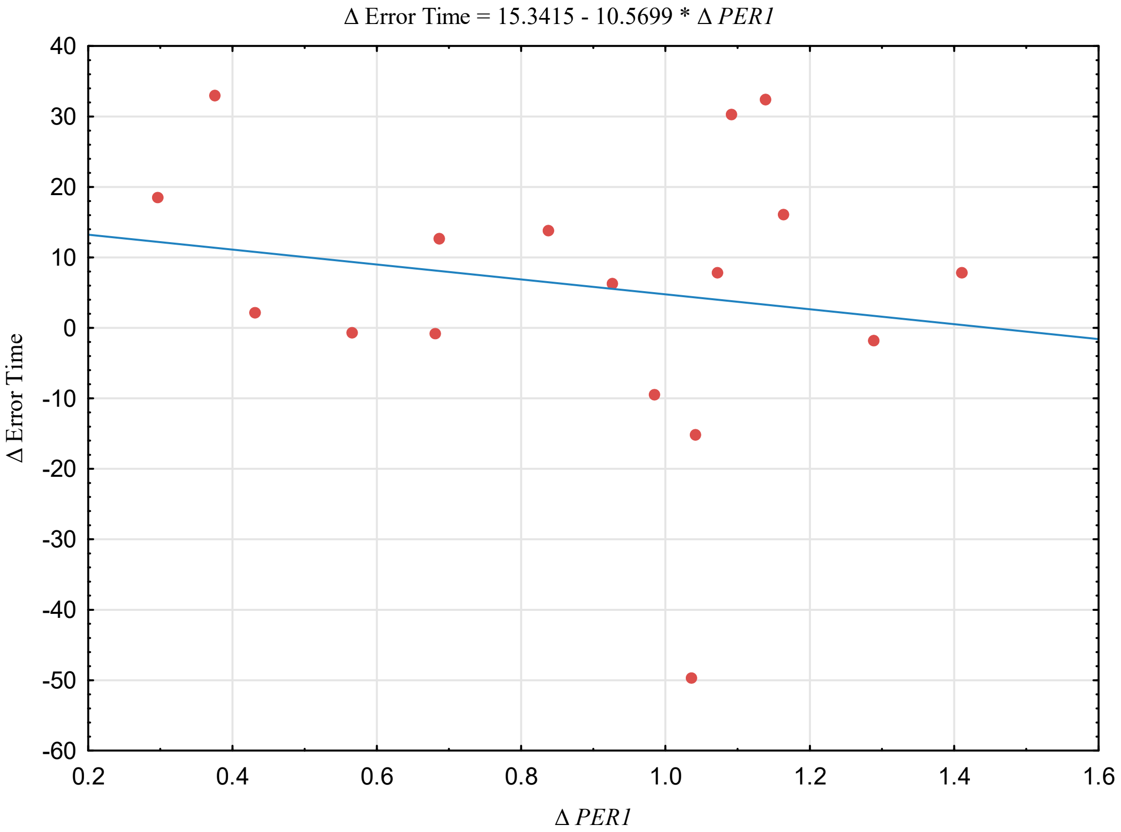

Abbreviations: PER1 - Period circadian regulator 1.

Figure S27. Scatterplot of Δ*CRY1* expression versus ΔBimanual Eye-Hand Coordination Test Task Duration in the entire study group.

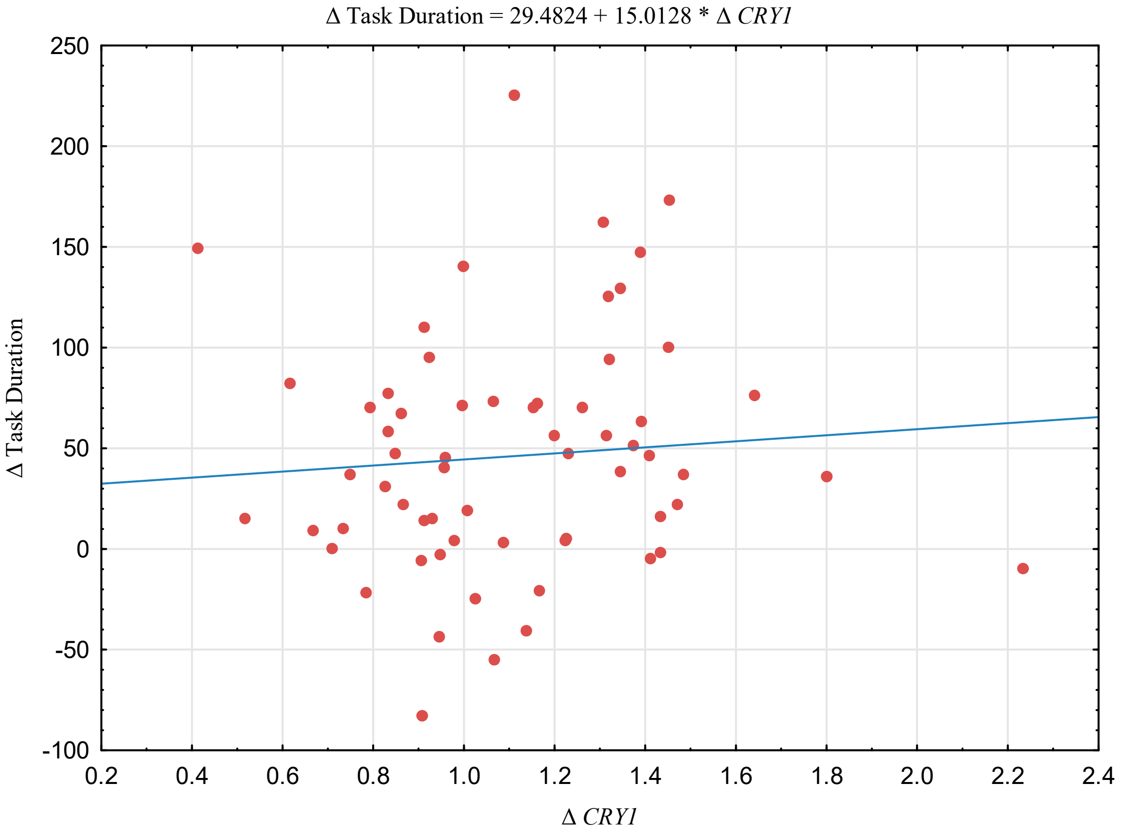

Abbreviations: CRY1 - Cryptochrome Circadian Regulator 1.

Figure S28. Scatterplot of Δ*CRY1* expression versus ΔBimanual Eye-Hand Coordination Test Error Time in the entire study group.

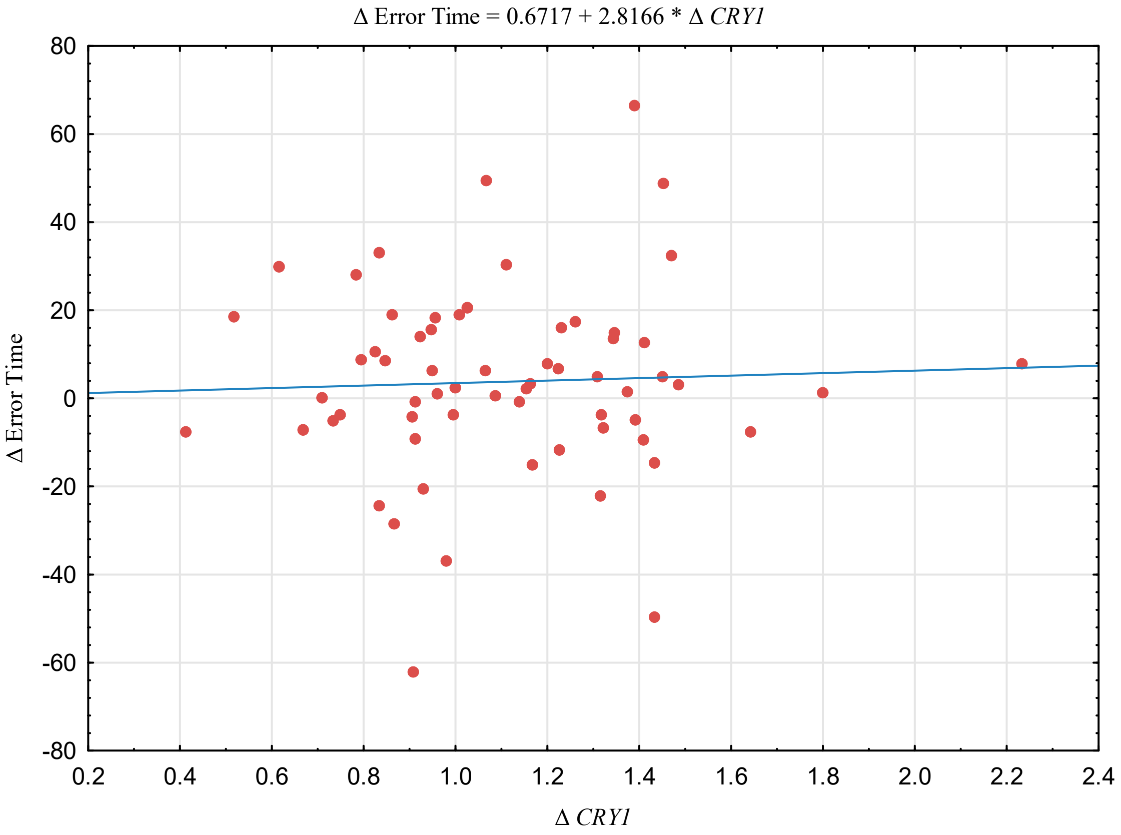

Abbreviations: CRY1 - Cryptochrome Circadian Regulator 1.

Figure S29. Scatterplot of Δ*CRY1* expression versus ΔBimanual Eye-Hand Coordination Test Error Count in the entire study group.

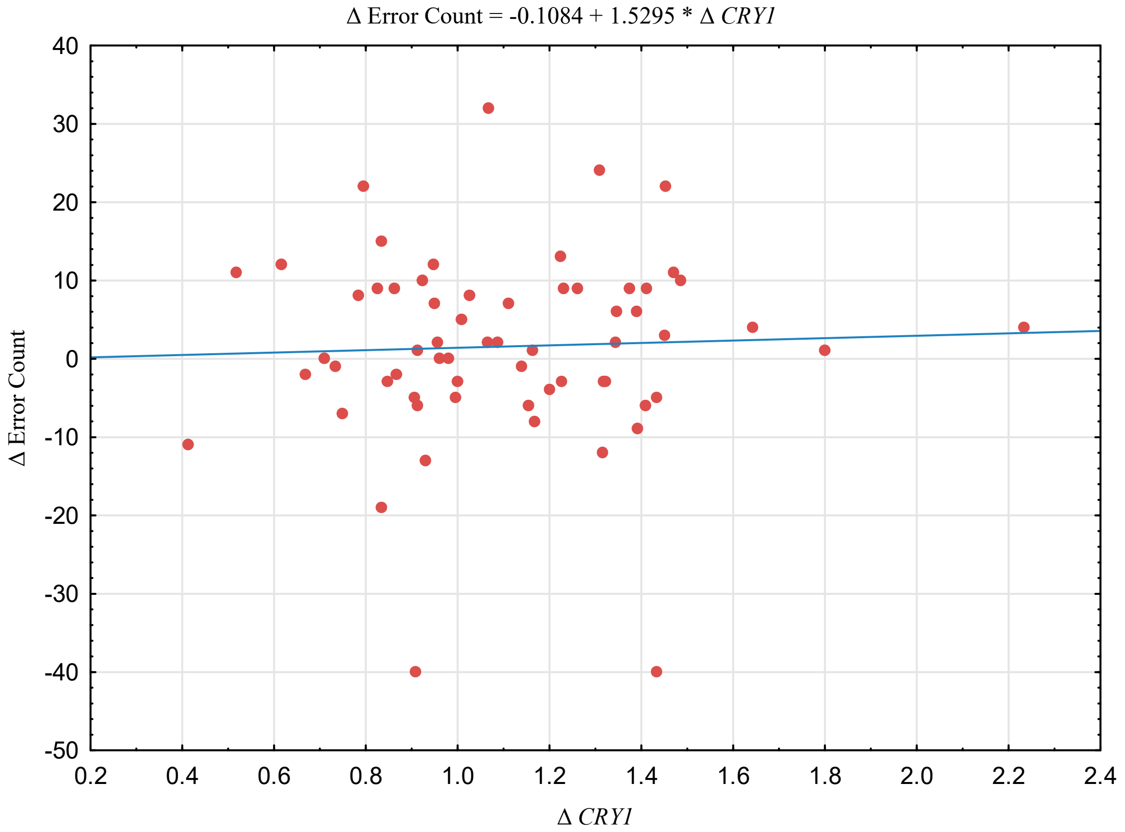

Abbreviations: CRY1 - Cryptochrome Circadian Regulator 1.

Figure S30. Scatterplot of Δ*CRY1* expression versus ΔBimanual Eye-Hand Coordination Test Task Duration in Responders.

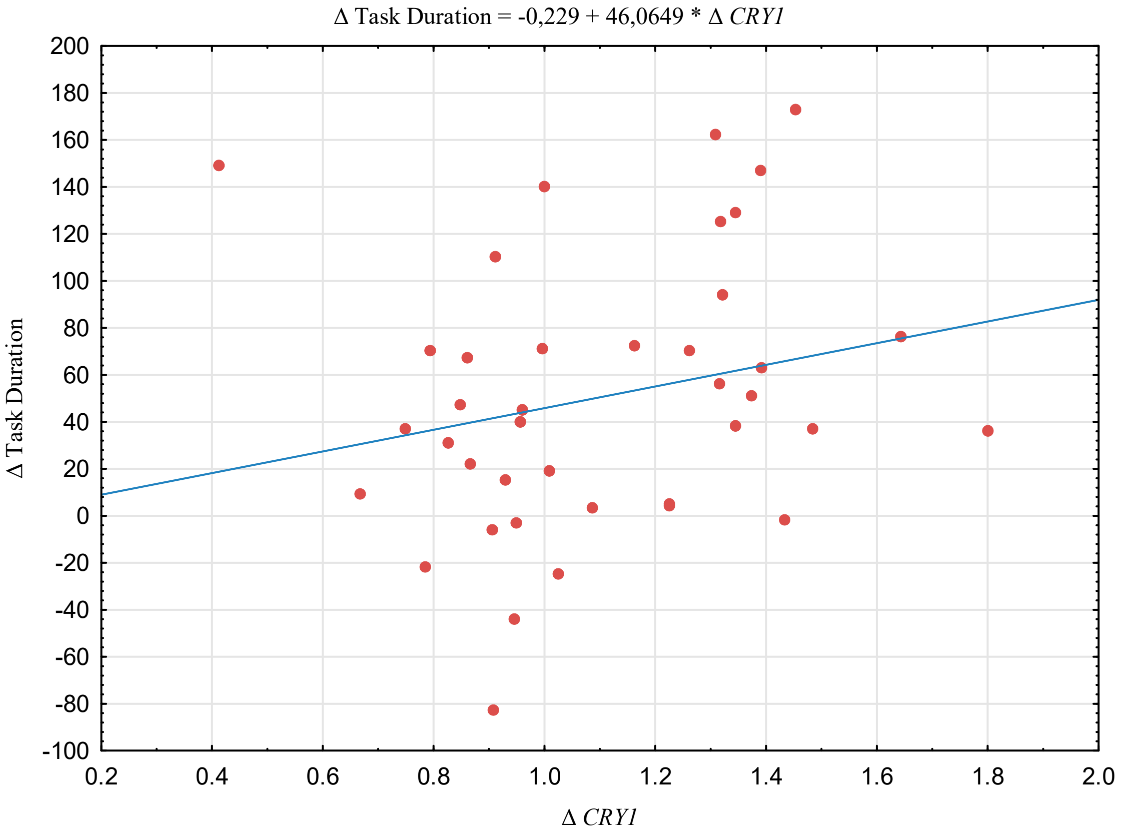

Abbreviations: CRY1 - Cryptochrome Circadian Regulator 1.

Figure S31. Scatterplot of Δ*CRY1* expression versus ΔBimanual Eye-Hand Coordination Test Error Time in Responders.

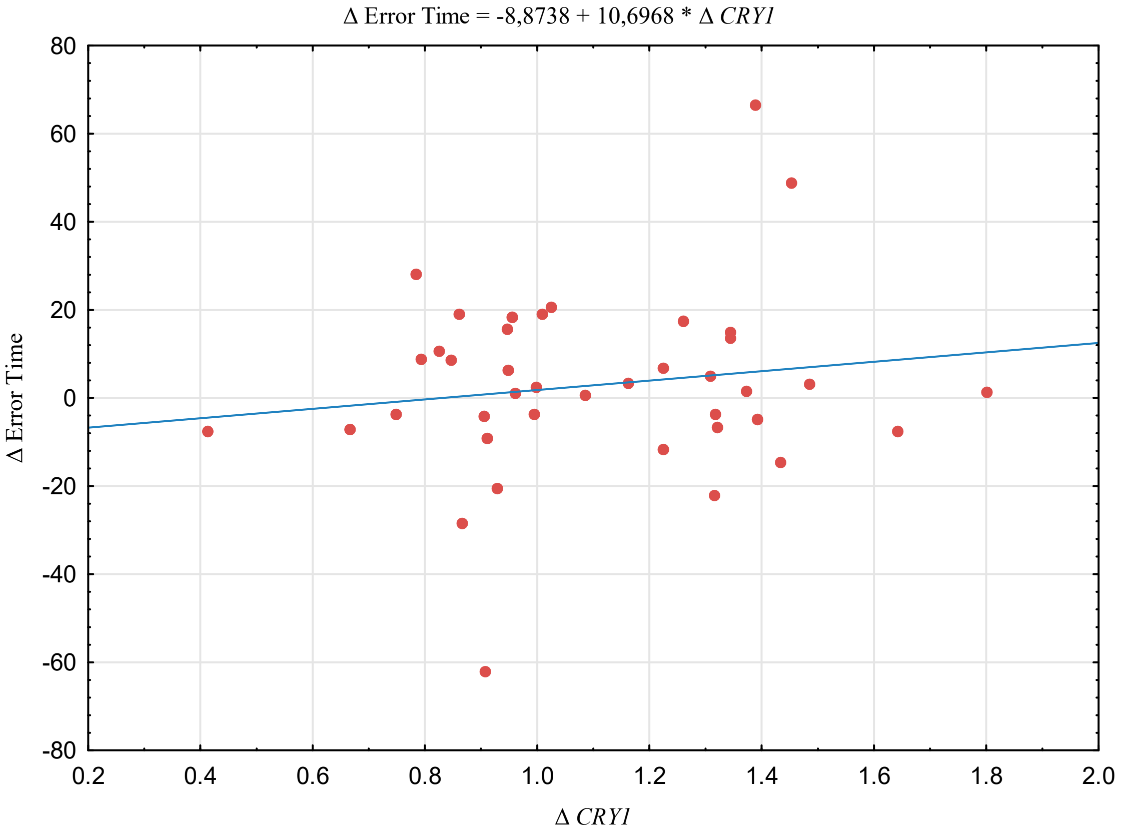

Abbreviations: CRY1 - Cryptochrome Circadian Regulator 1.

Figure S32. Scatterplot of Δ*CRY1* expression versus ΔBimanual Eye-Hand Coordination Test Error Count in Responders.

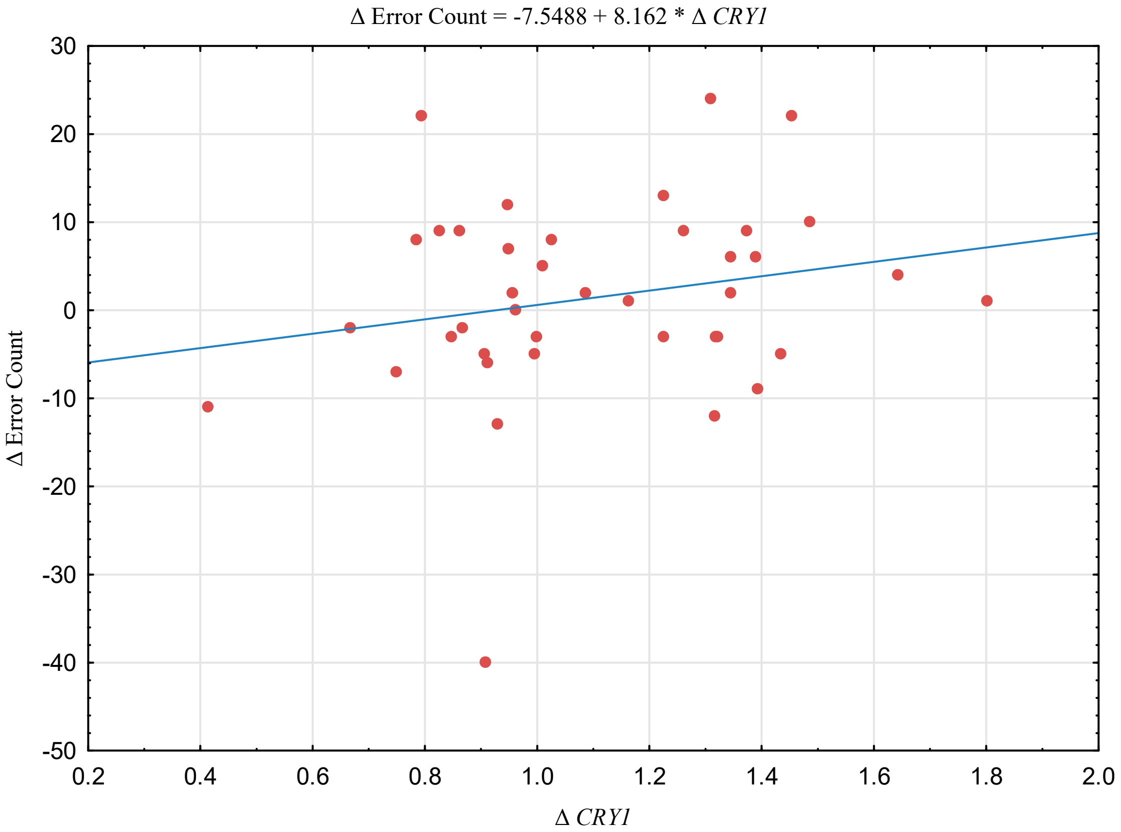

Abbreviations: CRY1 - Cryptochrome Circadian Regulator 1.

Figure S33. Scatterplot of Δ*CRY1* expression versus ΔBimanual Eye-Hand Coordination Test Task Duration in Non-Responders.

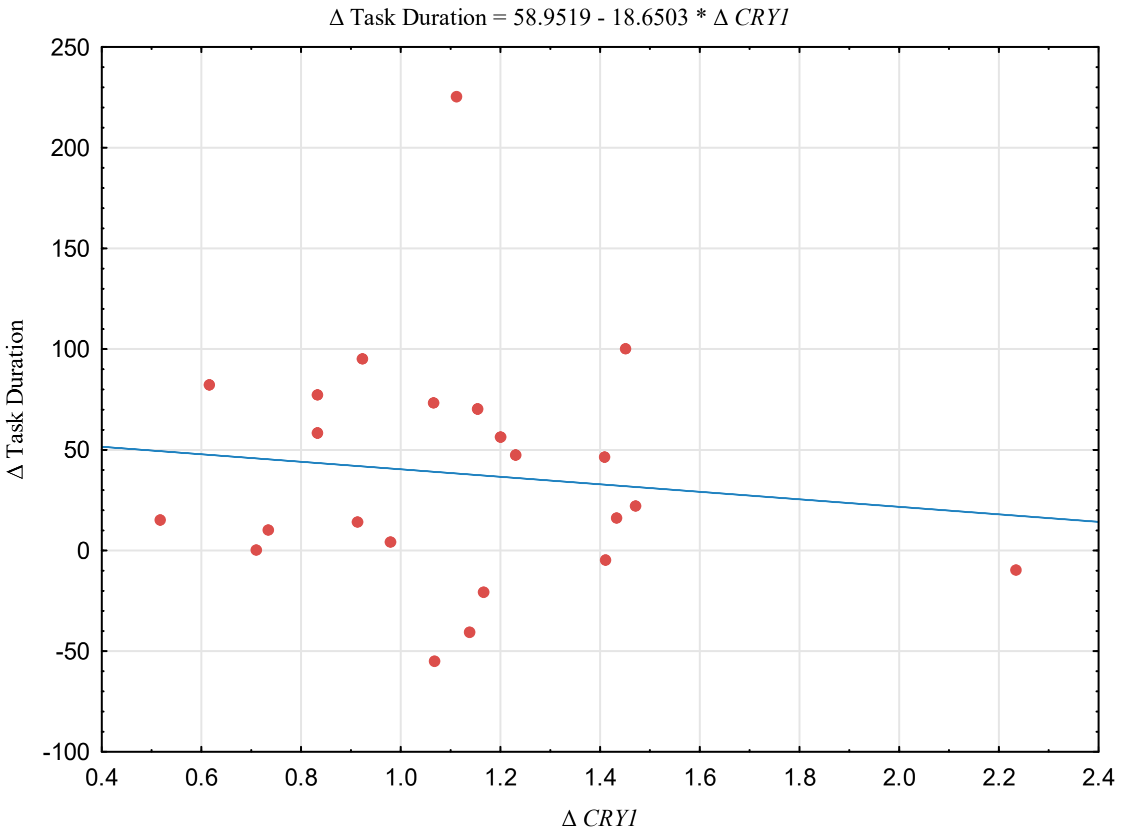

Abbreviations: CRY1 - Cryptochrome Circadian Regulator 1.

Figure S34. Scatterplot of Δ*CRY1* expression versus ΔBimanual Eye-Hand Coordination Test Error Time in Non-Responders

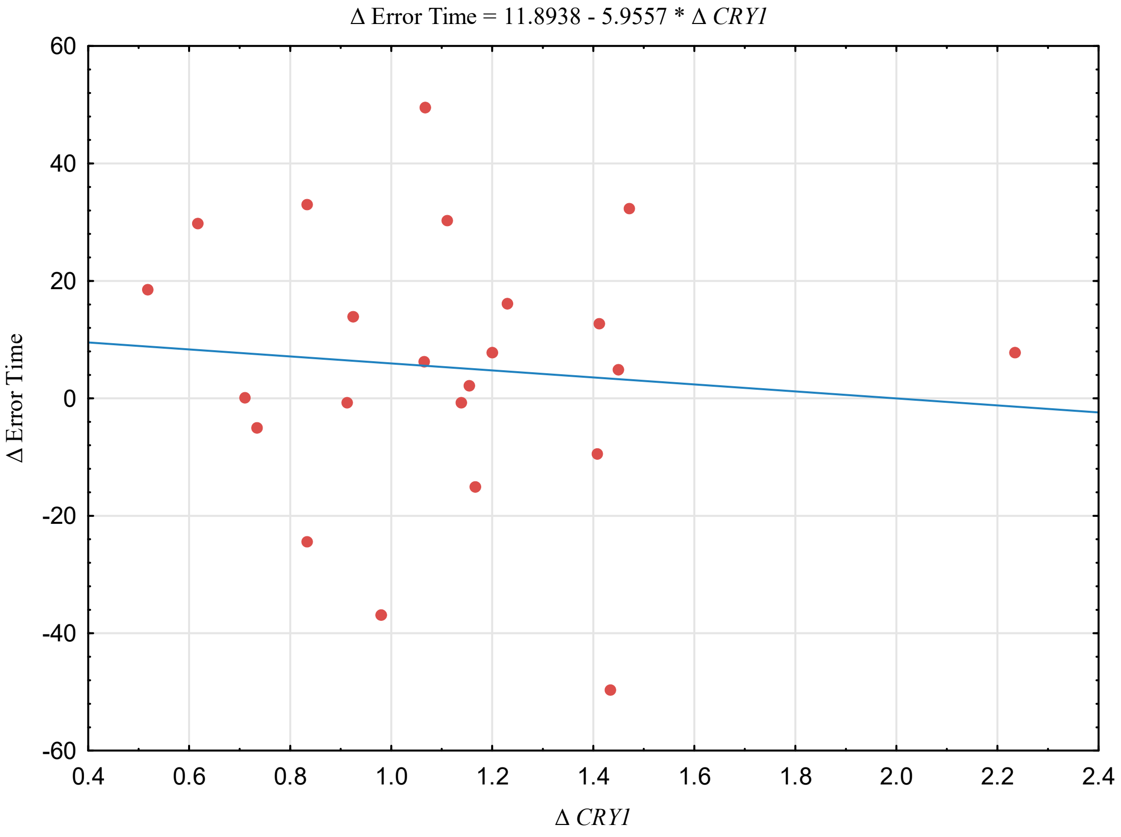

Abbreviations: CRY1 - Cryptochrome Circadian Regulator 1.

Figure S35. Scatterplot of Δ*CRY1* expression versus ΔBimanual Eye-Hand Coordination Test Error Count in Non-Responders.

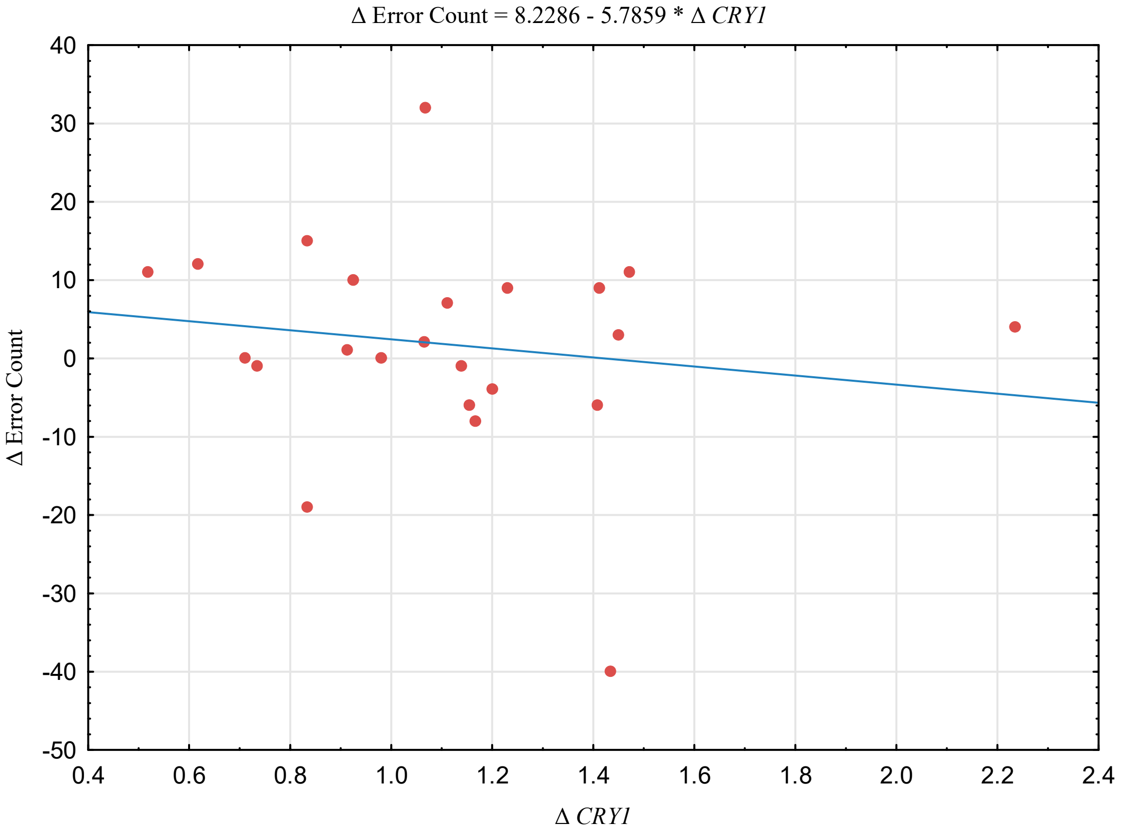

Abbreviations: CRY1 - Cryptochrome Circadian Regulator 1.

Figure S36. Scatterplot of Δ*NR1D1* expression versus ΔBimanual Eye-Hand Coordination Test Task Duration in the entire study group.

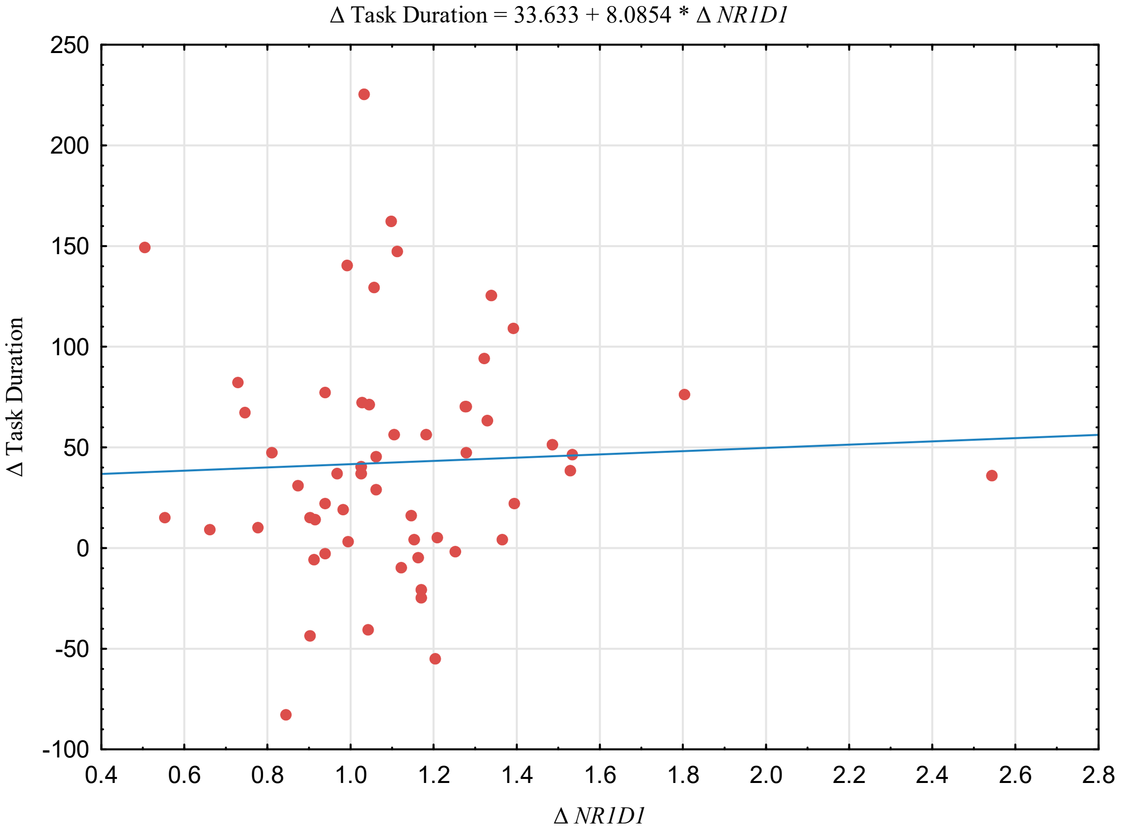

Abbreviations: NR1D1 - Nuclear Receptor Subfamily 1 Group D Member 1.

Figure S37. Scatterplot of Δ*NR1D1* expression versus ΔBimanual Eye-Hand Coordination Test Error Time in the entire study group.

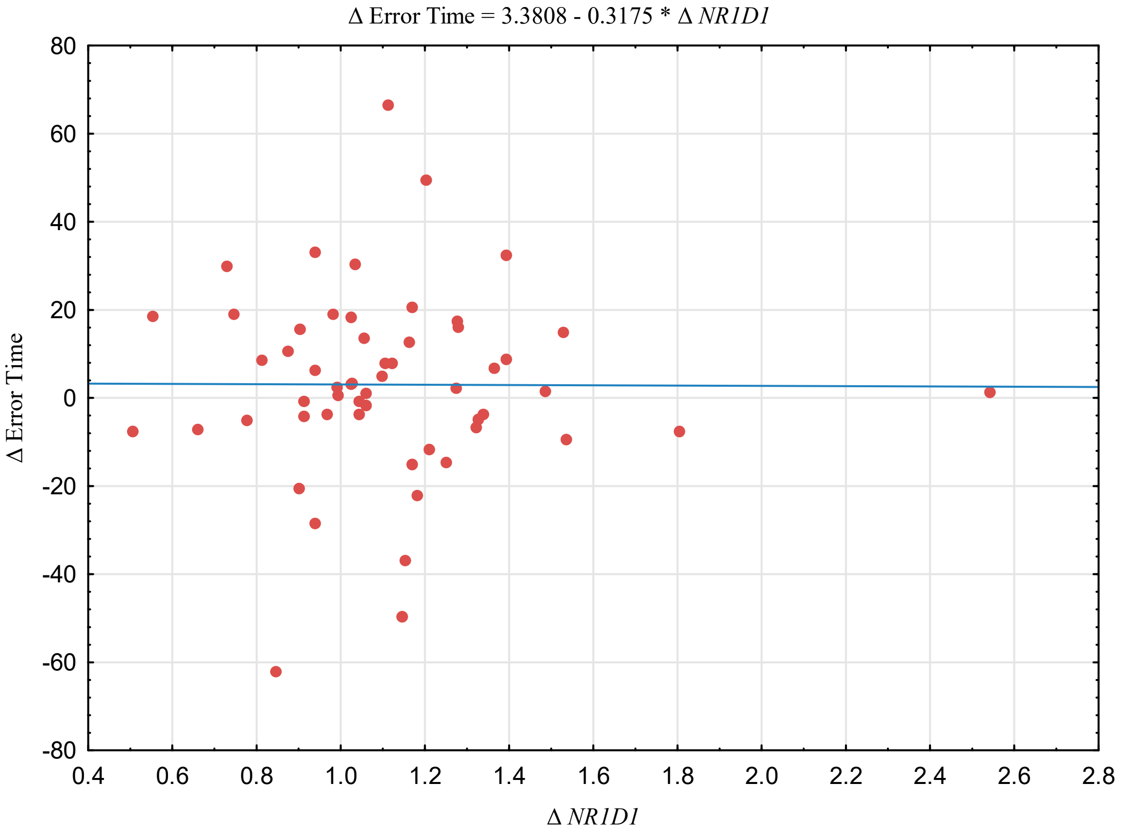

Abbreviations: NR1D1 - Nuclear Receptor Subfamily 1 Group D Member 1.

Figure S38. Scatterplot of Δ*NR1D1* expression versus ΔBimanual Eye-Hand Coordination Test Error Count in the entire study group.

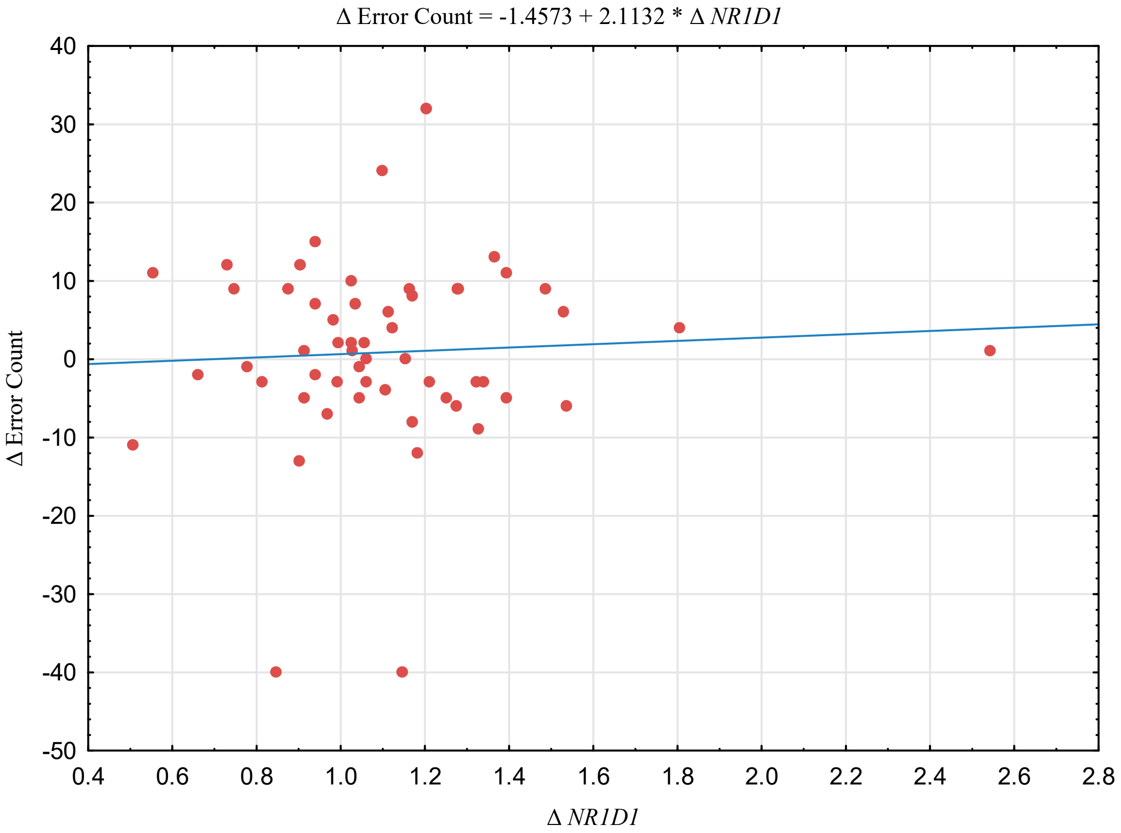

Abbreviations: NR1D1 - Nuclear Receptor Subfamily 1 Group D Member 1.

Figure S39. Scatterplot of Δ*NR1D1* expression versus ΔBimanual Eye-Hand Coordination Test Task Duration in Responders.

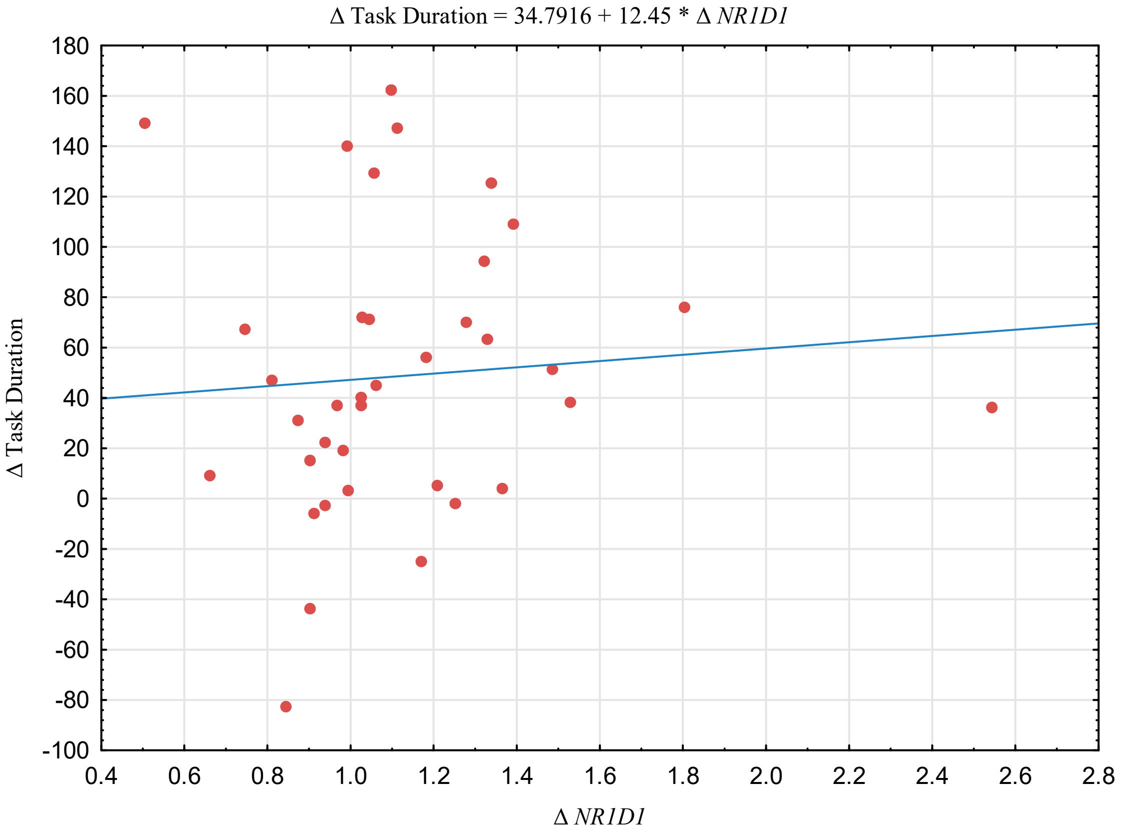

Abbreviations: NR1D1 - Nuclear Receptor Subfamily 1 Group D Member 1.

Figure S40. Scatterplot of Δ*NR1D1* expression versus ΔBimanual Eye-Hand Coordination Test Error Time in Responders.

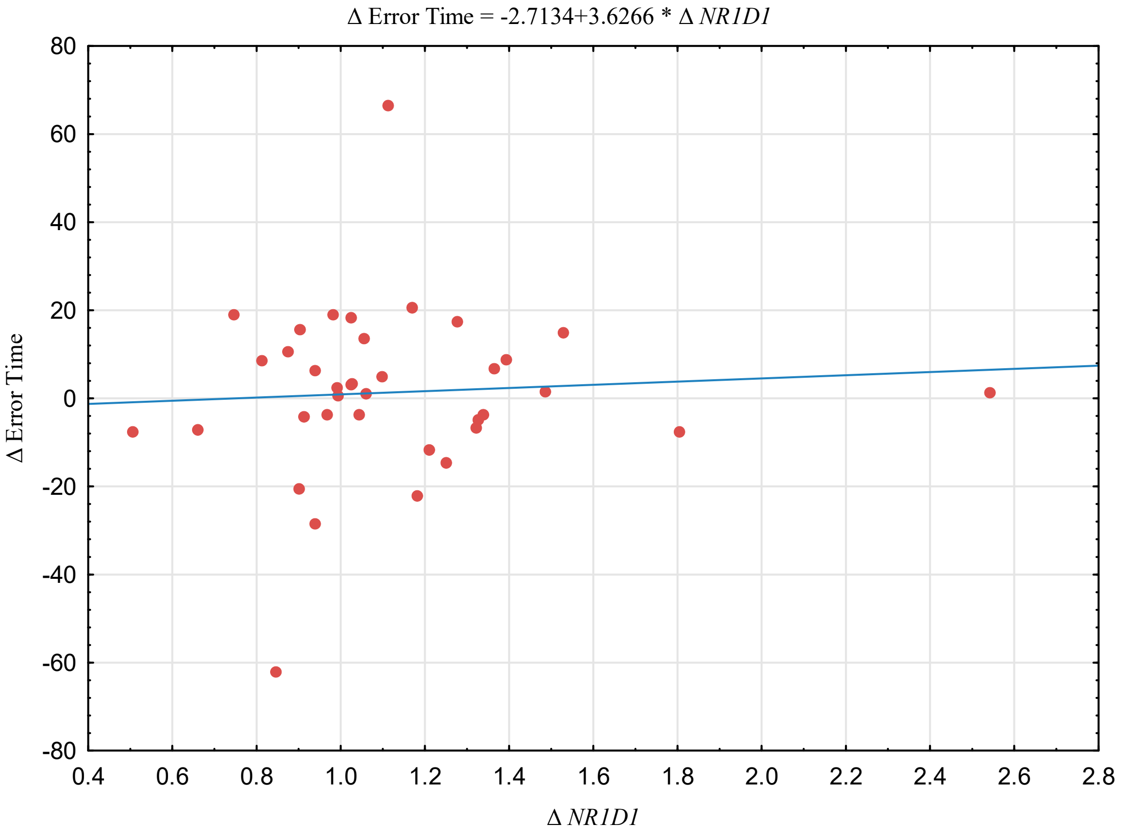

Abbreviations: NR1D1 - Nuclear Receptor Subfamily 1 Group D Member 1.

Figure S41. Scatterplot of Δ*NR1D1* expression versus ΔBimanual Eye-Hand Coordination Test Error Count in Responders.

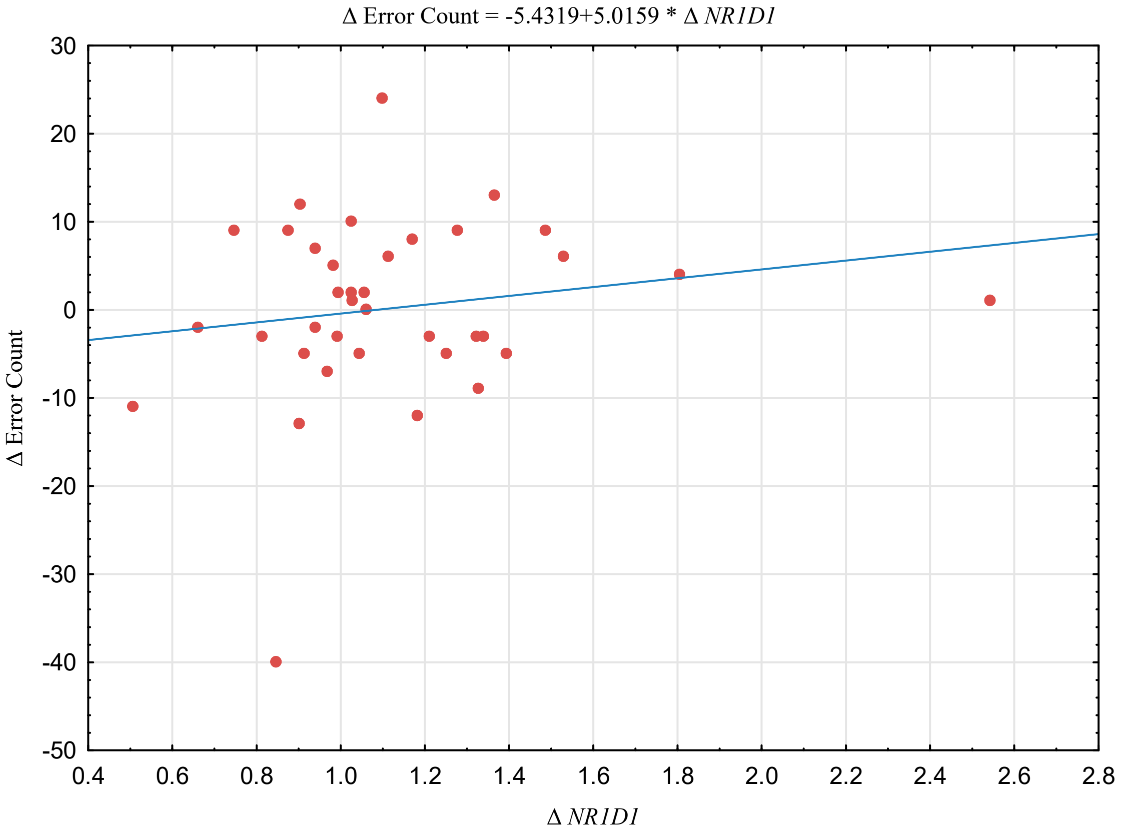

Abbreviations: NR1D1 - Nuclear Receptor Subfamily 1 Group D Member 1.

Figure S42. Scatterplot of Δ*NR1D1* expression versus ΔBimanual Eye-Hand Coordination Test Task Duration in Non-Responders.

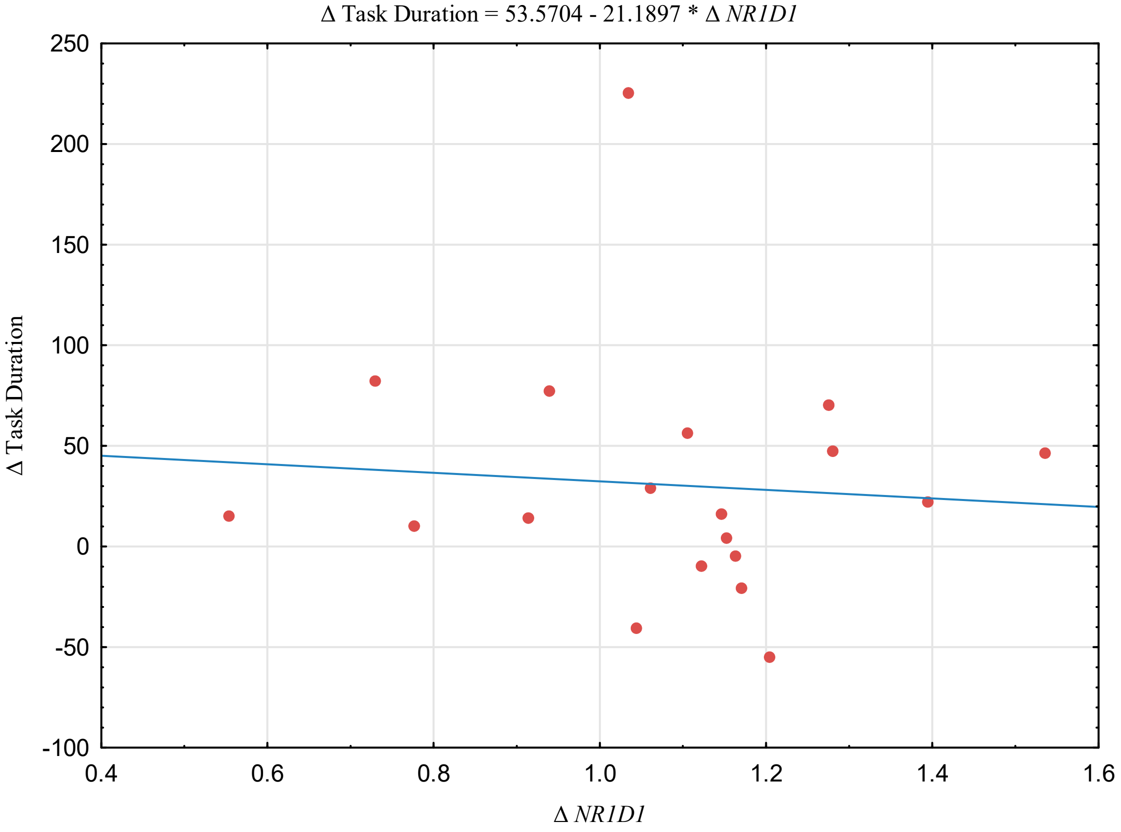

Abbreviations: NR1D1 - Nuclear Receptor Subfamily 1 Group D Member 1.

Figure S43. Scatterplot of Δ*NR1D1* expression versus ΔBimanual Eye-Hand Coordination Test Error Time in Non-Responders.

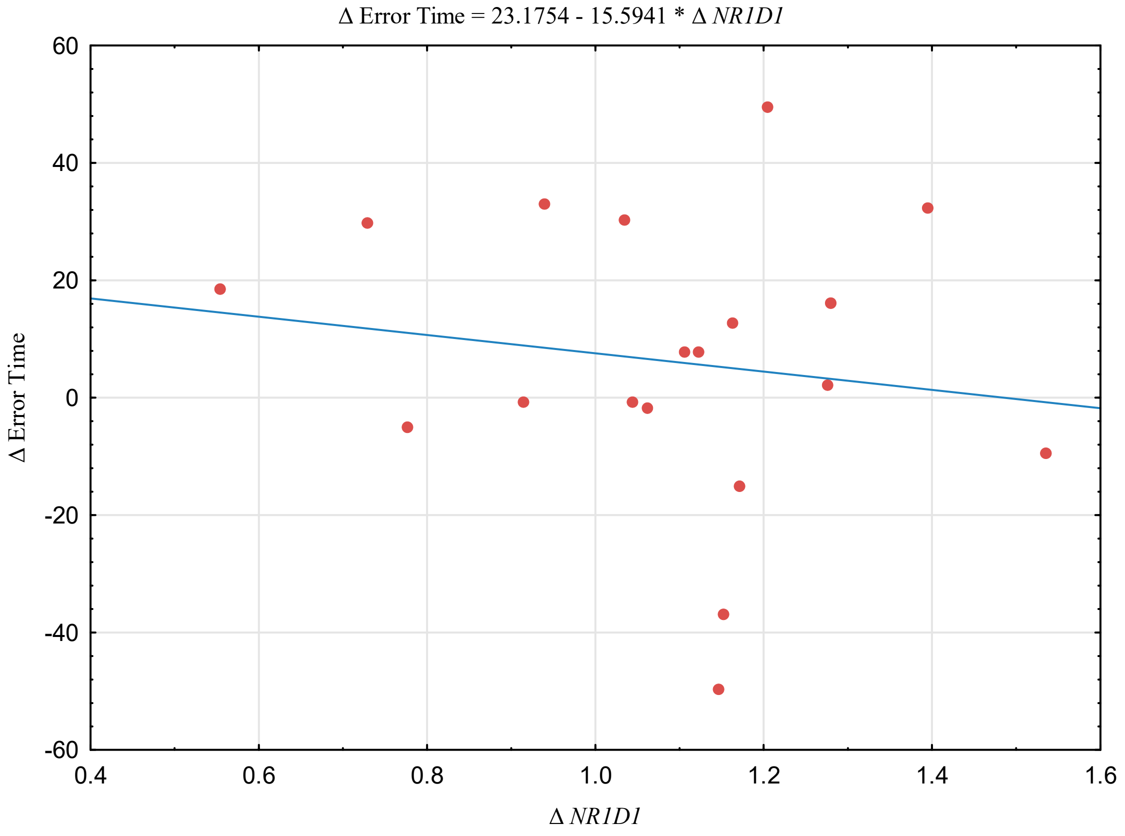

Abbreviations: NR1D1 - Nuclear Receptor Subfamily 1 Group D Member 1.

Figure S44. Scatterplot of Δ*NR1D1* expression versus ΔBimanual Eye-Hand Coordination Test Error Count in Non-Responders.

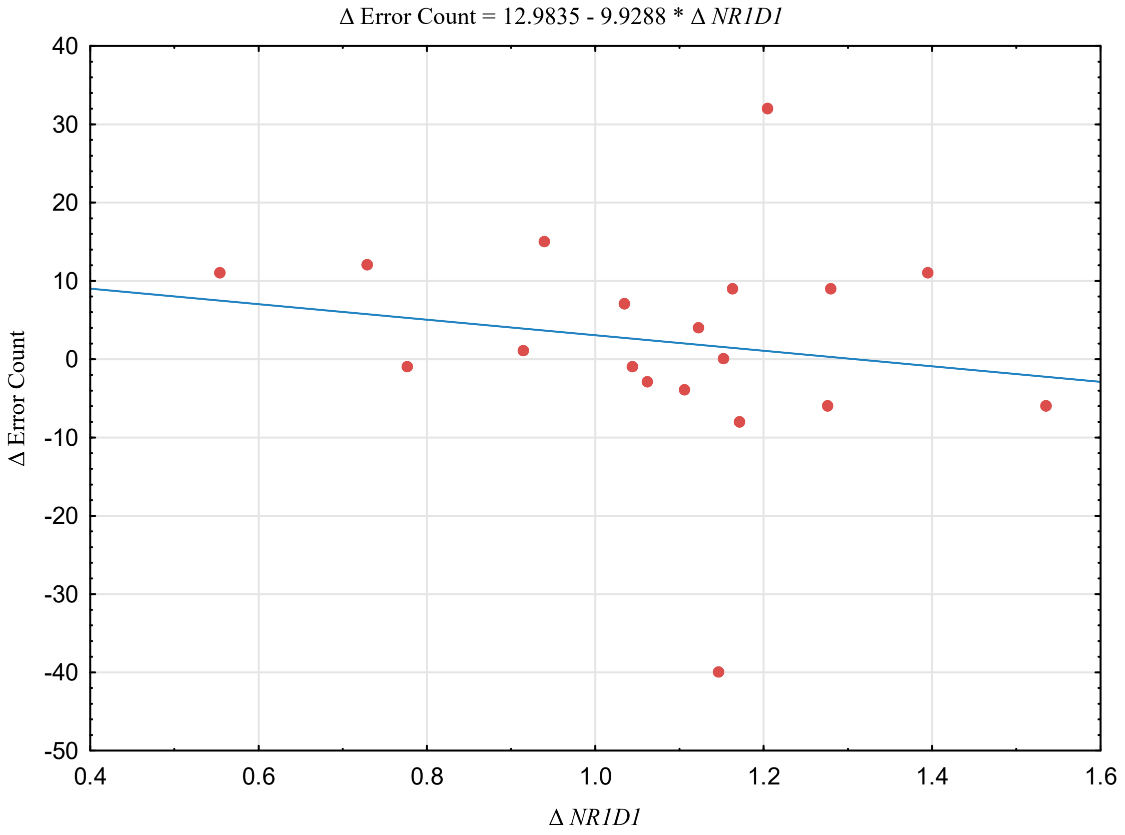

Abbreviations: NR1D1 - Nuclear Receptor Subfamily 1 Group D Member 1.

Figure S45. Scatterplot of Δ*NPAS2* expression versus ΔBimanual Eye-Hand Coordination Test Task Duration in the entire study group.

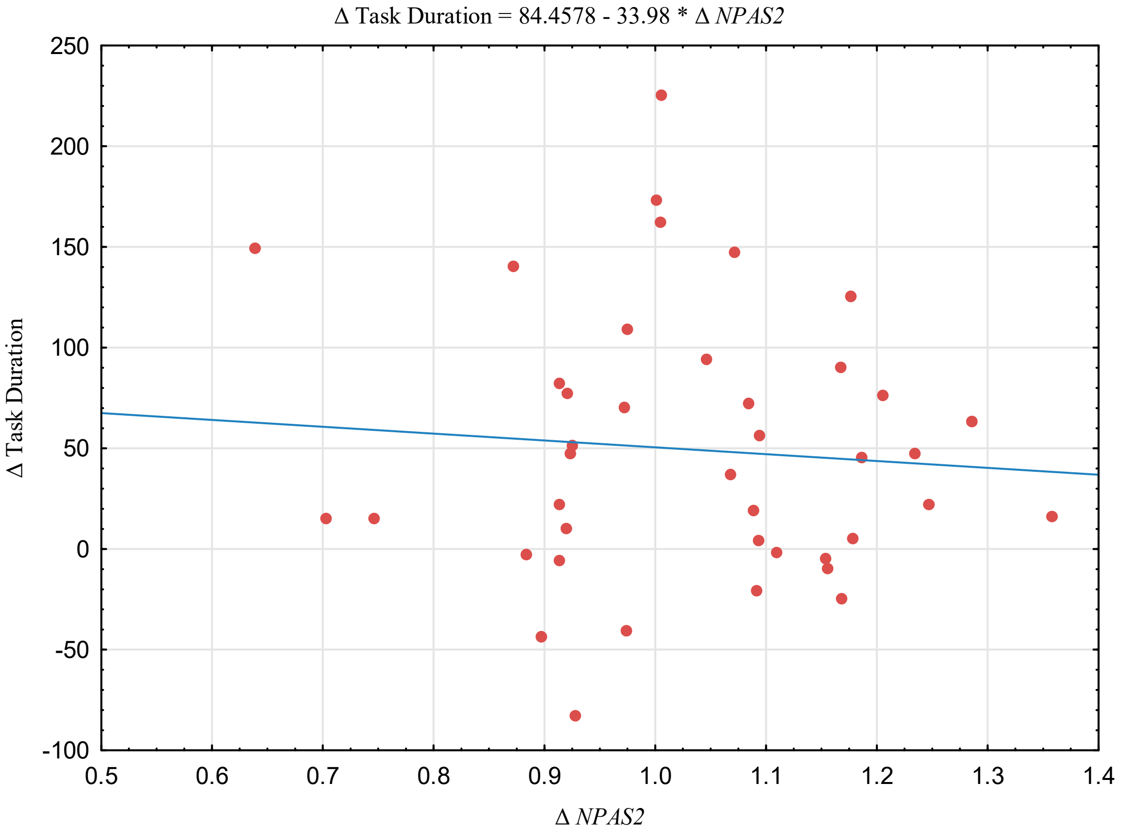

Abbreviations: NPAS2 - Neuronal PAS domain protein 2.

Figure S46. Scatterplot of Δ*NPAS2* expression versus ΔBimanual Eye-Hand Coordination Test Error Time in the entire study group.

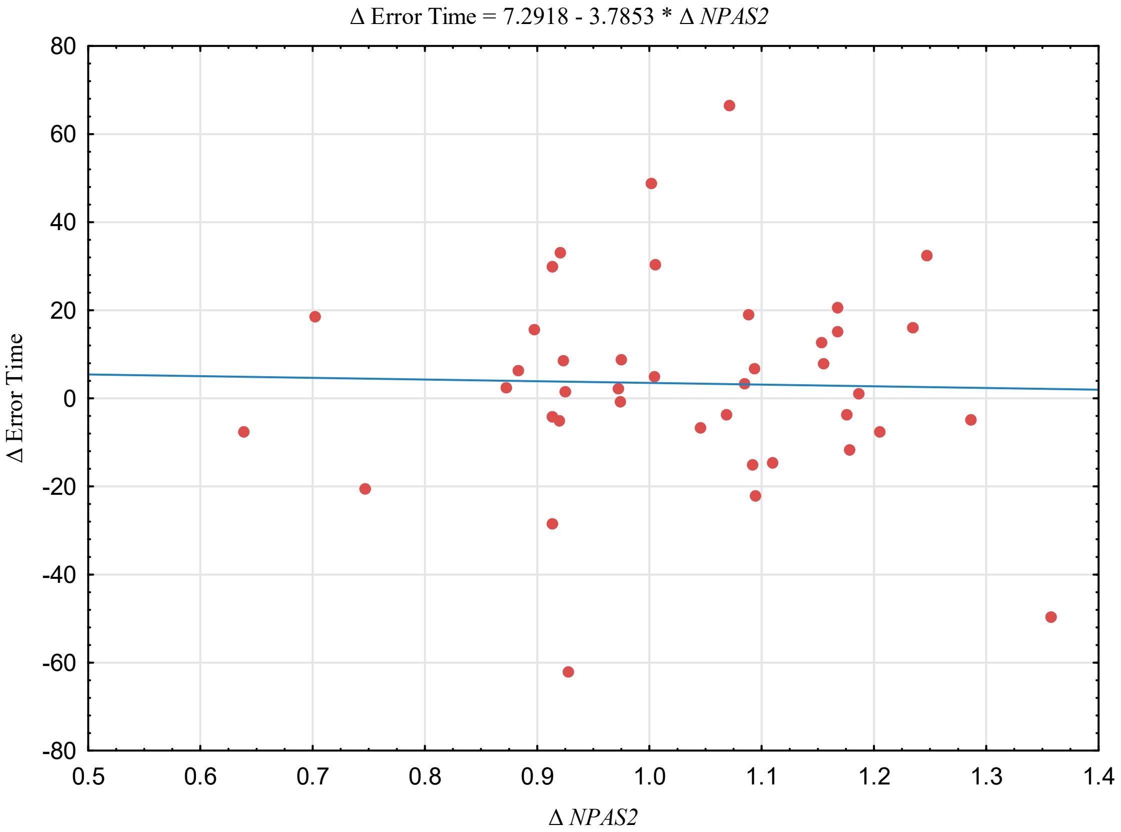

Abbreviations: NPAS2 - Neuronal PAS domain protein 2.

Figure S47. Scatterplot of Δ*NPAS2* expression versus ΔBimanual Eye-Hand Coordination Test Error Count in the entire study group.

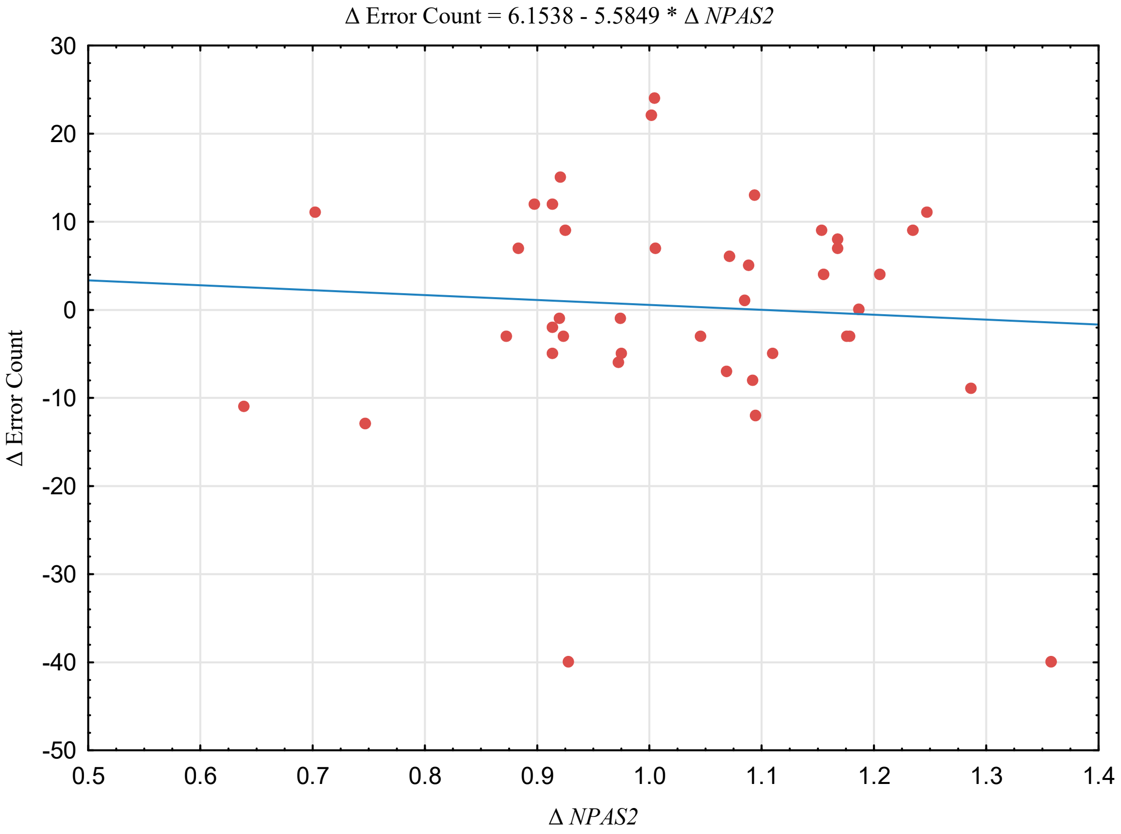

Abbreviations: NPAS2 - Neuronal PAS domain protein 2.

Figure S48. Scatterplot of Δ*NPAS2* expression versus ΔBimanual Eye-Hand Coordination Test Task Duration in Responders.

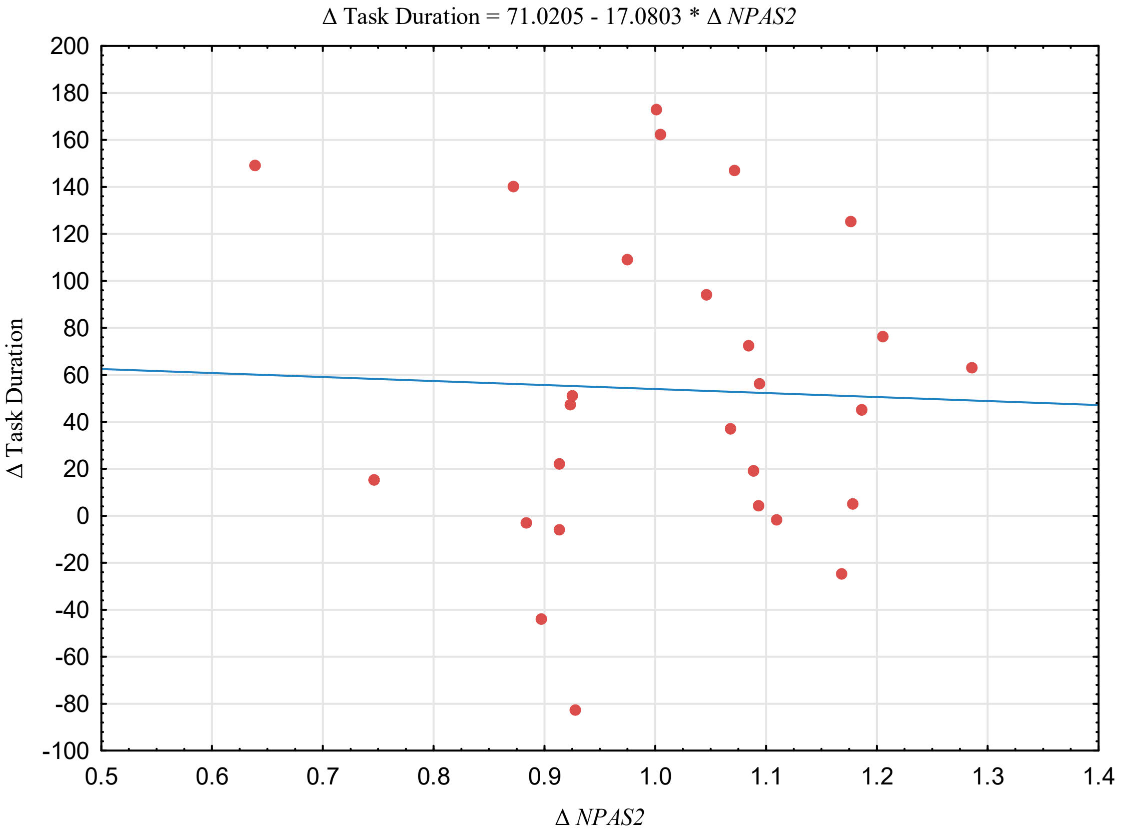

Abbreviations: NPAS2 - Neuronal PAS domain protein 2.

Figure S49. Scatterplot of Δ*NPAS2* expression versus ΔBimanual Eye-Hand Coordination Test Error Time in Responders.

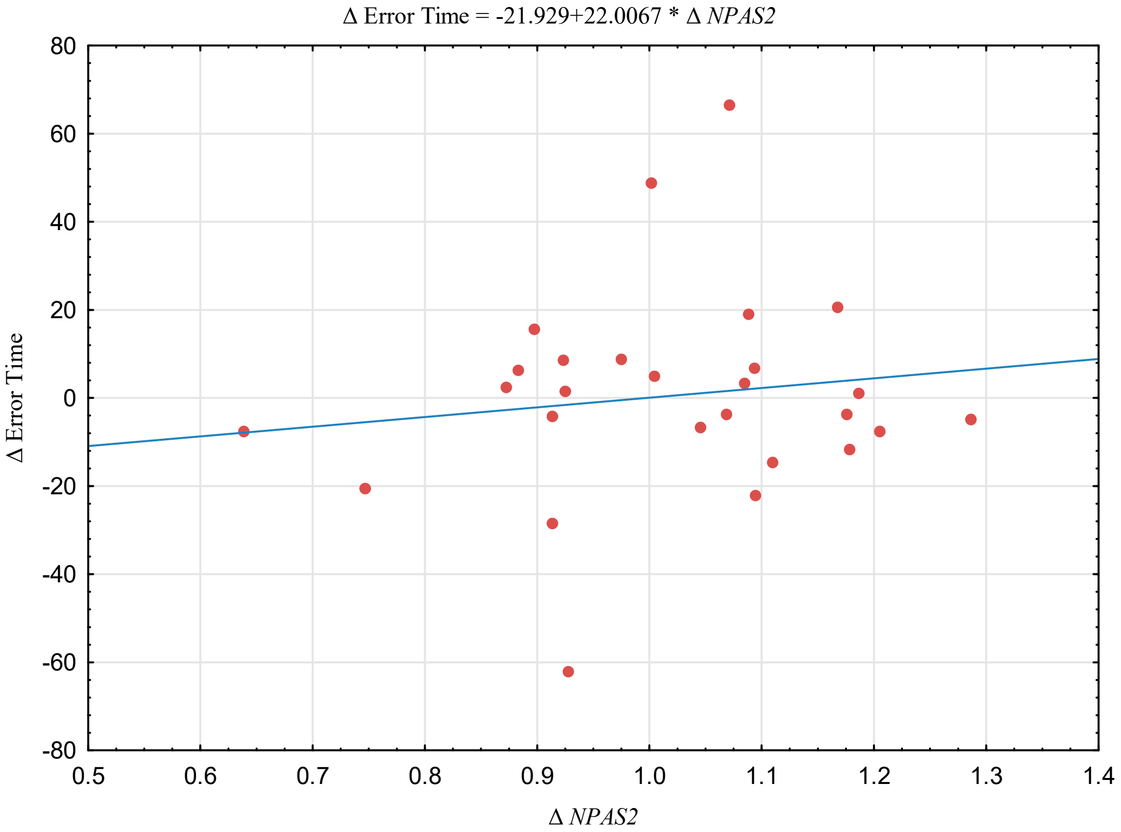

Abbreviations: NPAS2 - Neuronal PAS domain protein 2.

Figure S50. Scatterplot of Δ*NPAS2* expression versus ΔBimanual Eye-Hand Coordination Test Error Count in Responders.

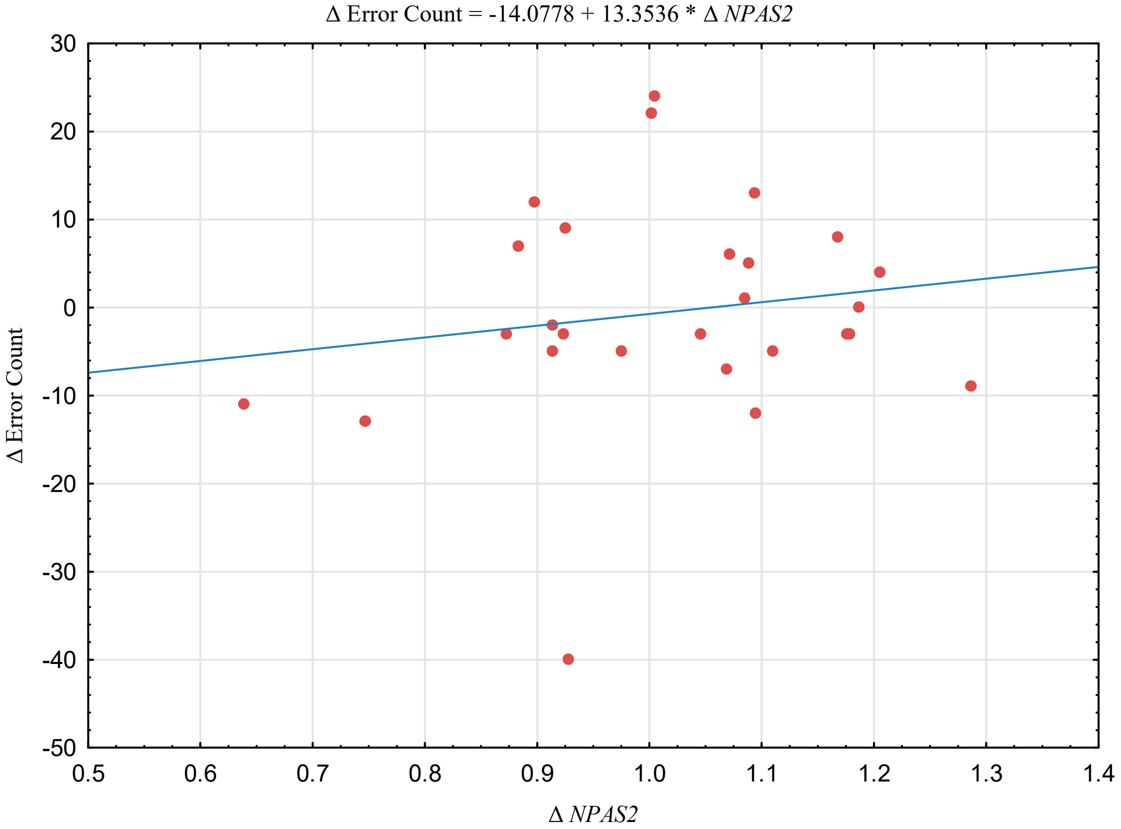

Abbreviations: NPAS2 - Neuronal PAS domain protein 2.

Figure S51. Scatterplot of Δ*NPAS2* expression versus ΔBimanual Eye-Hand Coordination Test Task Duration in Non-Responders.

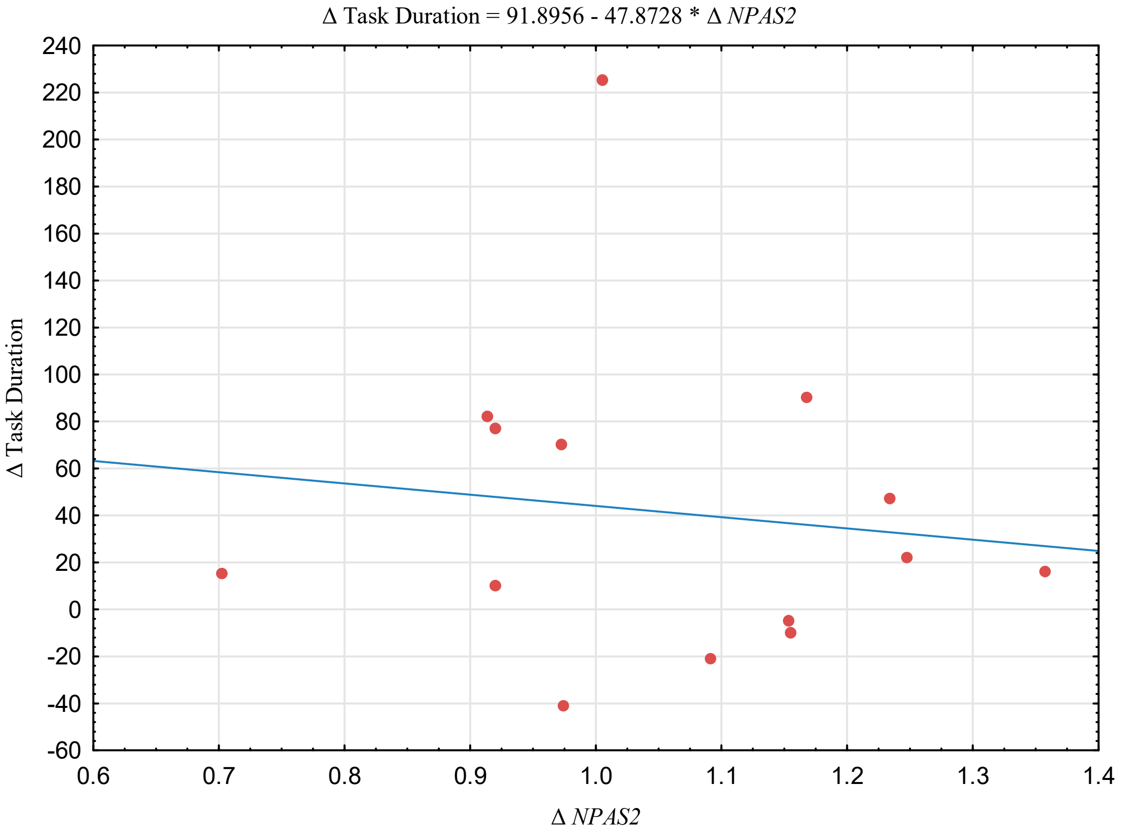

Abbreviations: NPAS2 - Neuronal PAS domain protein 2.

Figure S52. Scatterplot of Δ*NPAS2* expression versus ΔBimanual Eye-Hand Coordination Test Error Time in Non-Responders.

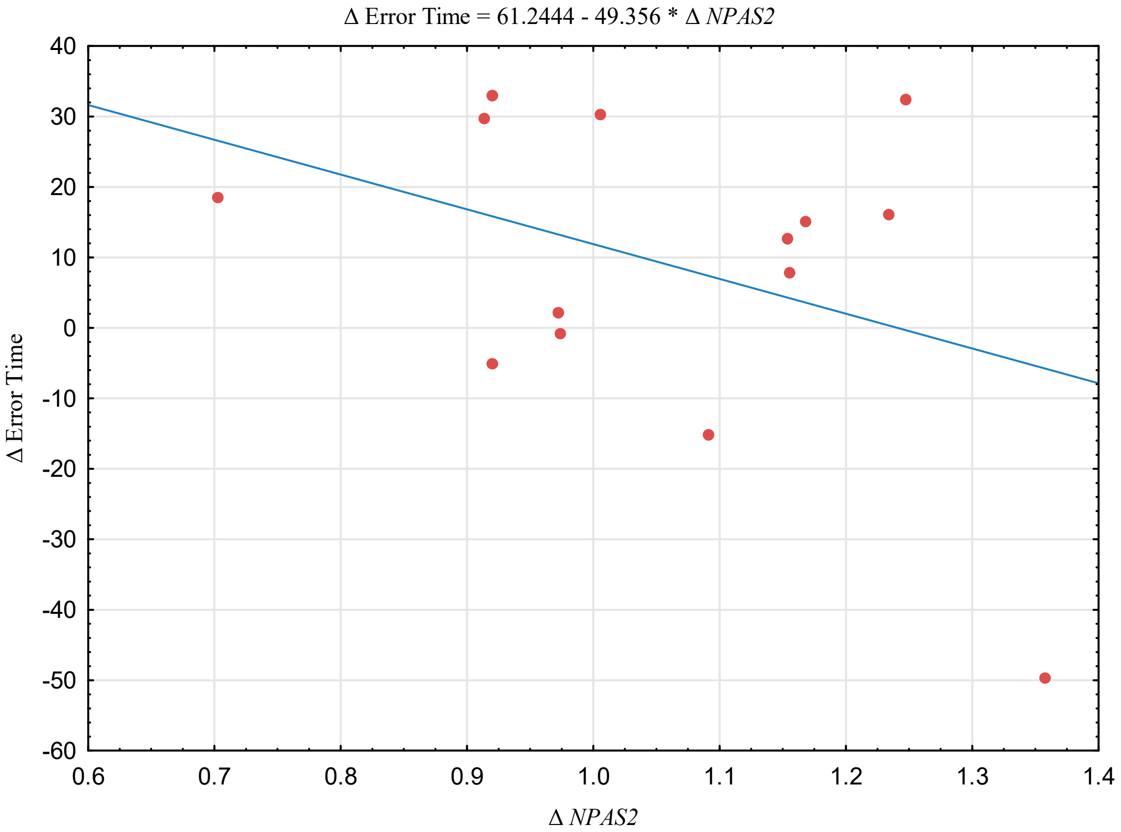

Abbreviations: NPAS2 - Neuronal PAS domain protein 2.

Figure S53. Scatterplot of Δ*NPAS2* expression versus ΔBimanual Eye-Hand Coordination Test Error Count in Non-Responders.

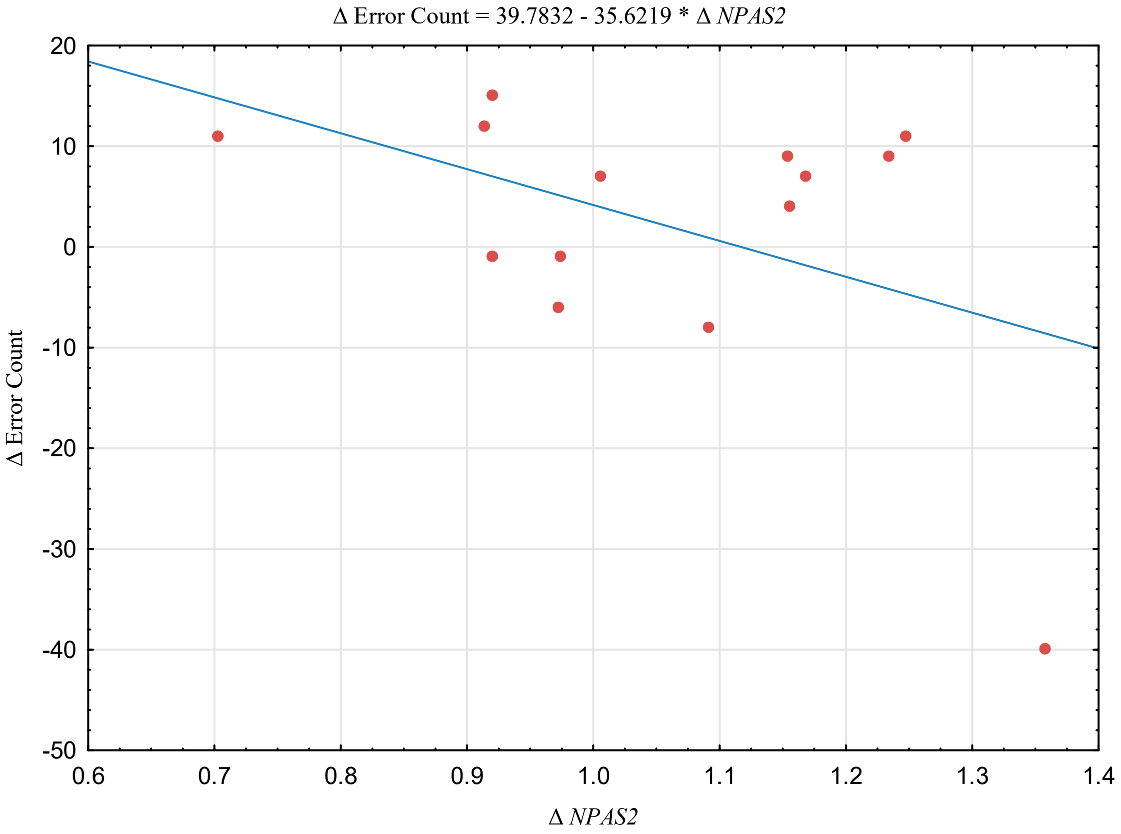

Abbreviations: NPAS2 - Neuronal PAS domain protein 2.
